# SO_2_F_2_-mediated transformation of 2'-hydroxyacetophenones to benzo-oxetes

**DOI:** 10.3762/bjoc.15.95

**Published:** 2019-04-25

**Authors:** Revathi Lekkala, Ravindar Lekkala, Balakrishna Moku, K P Rakesh, Hua-Li Qin

**Affiliations:** 1 State Key Laboratory of Silicate Materials for Architectures; School of Chemistry, Chemical Engineering and Life Science, Wuhan University of Technology, 205 Luoshi Road, Wuhan 430070, China

**Keywords:** benzo-oxetes, 2'-hydroxyacetophenones, sulfuryl fluoride

## Abstract

A catalyst-free novel and efficient methodology for the challenging synthesis of benzo-oxetes from 2'-hydroxyacetophenones mediated by sulfuryl fluoride (SO_2_F_2_) gas has been realized. The combination of 2'-hydroxyacetophenones and SO_2_F_2_ furnishes synthetically challenging benzo-oxetanes in moderate to excellent yields. The highlight of this work is the design and synthesis of strained four-membered oxete rings.

## Introduction

Oxetanes are versatile elements in drug discovery and synthesis [1–2], and represent important moieties in some natural products and biological active molecules [3]. The synthesis and chemistry of oxetanes have been reviewed [1–2,4–5]. In medicinal chemistry, oxetanes have remained neglected units for many years since the first parent structure has been reported by Reboul in 1878 [6]. As strained cyclic ethers oxetanes exhibit an attractive combination of stable patterns for medicinal chemistry and reactivity for further synthesis [4]. These characteristics formulate them a captivating pattern for a rising scope of applications in the chemical sciences. Recently, oxetanes emerged as small, stable, attractive, and less lipophilic molecular units in drug discovery [7–8]. In view of the broad applicability of oxetanes in materials sciences, organic synthesis, and medicinal chemistry [7–15], the progress of synthetic routes for the formation of a range of oxetanes is highly desirable.

**Scheme 1:**
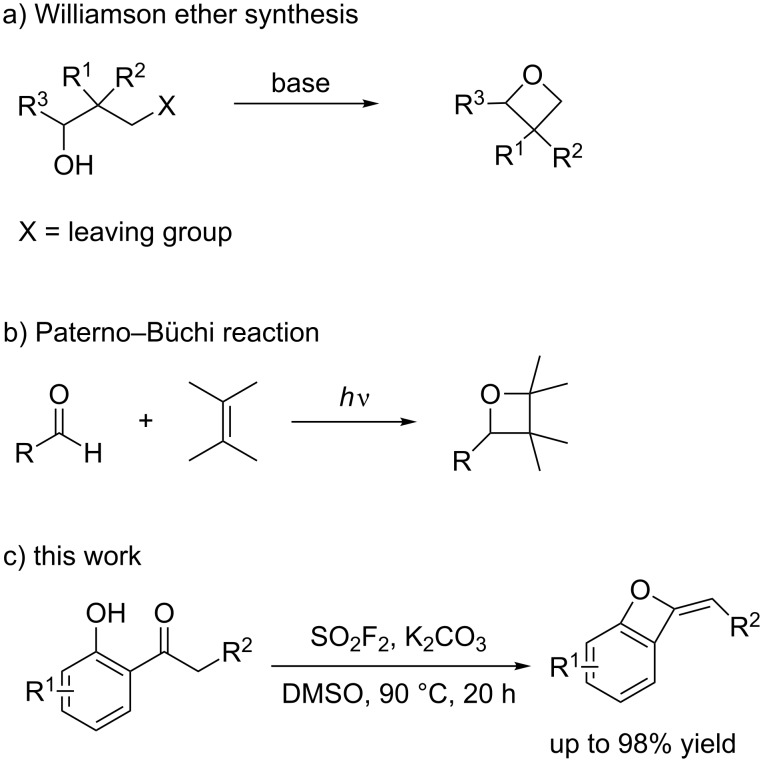
Synthetic pathways towards oxetanes and benzo-oxetes.

However, the intrinsic ring strain in oxetanes makes cyclization a basic challenge, moreover, the kinetics of cyclization to make four-membered ring ethers are considerably slower than those of six, five, and three-membered ring compounds [16]. Therefore, the formation of oxetanes with satisfactory yields from the functionalized acyclic precursors generally requires good leaving groups and anions. A large number of synthetic methods has been described for the formation of oxetane derivatives and among them, two common strategies are widely applied. The first strategy engages an intramolecular etherification reaction, which is called Williamson ether synthesis (Scheme 1a). This method was first utilized to synthesize oxetanes in 1878 and has remained as general tool in the synthesis of complex oxetane-containing compounds since then [17–20]. The second strategy involves a [2 + 2] cycloaddition that furnishes substituted oxetanes rapidly, for example Paterno–Büchi reaction (Scheme 1b) [21]. However, the recognition of facial selectivity control in [2 + 2] cycloaddition is not an easy task.

The availability of various methods for the synthesis of oxetanes and their strained nature has afforded opportunities for the discovery of new transformations. Previous reports on the synthesis of substituted oxetanes from ketones described the requirement of chiral reagents or catalysts [11–14,22–24]. Gaseous sulfuryl fluoride (SO_2_F_2_) widely has been utilized as a fumigant for more than five decades [25–26], and only recently it has attracted significant attention as an organic synthetic reagent. SO_2_F_2_ is a cheap and relatively stable gas (up to 400 °C when dry) and a highly reactive electrophile [27–29]. Under basic conditions, SO_2_F_2_ hydrolyzes rapidly into fluoride and fluorosulfate ions. In continuation of our research on the application of SO_2_F_2_ for the transformation of organic compounds [30–37], we herein report a novel strategy for the synthesis of benzo-oxete derivatives from ketones using the inexpensive and abundant SO_2_F_2_ through a catalyst-free cascade method (Scheme 1c).

## Results and Discussion

Initially, we screened various reaction conditions (see Supporting Information File 1) using 1-(2-hydroxyphenyl)ethanone (**1a**) as a model substrate. As illustrated in Table 1, the desired product **2a** was produced in 50–76% yield when different inorganic bases (3 equiv) were used in DMSO at 90 °C under a SO_2_F_2_ atmosphere (balloon) for 15 h (Table 1, entries 1–4). The use of K_2_CO_3_ provided a significantly higher product yield than the other bases tested (Table 1, entry 5) under the same reaction conditions. When the reaction time was decreased from 15 to 5 h, the yield of the desired product dropped from 90% to 76% (Table 1, entry 6 vs entry 5). On the other hand, increasing the reaction time to 18 h led to an increased product yield (from 90% to 94%, Table 1, entry 7 vs entry 5) and after 20 h reaction time a maximum yield of 98% was achieved (Table 1, entry 8). Next, screening of the reaction temperature revealed that lowering the temperature from 90 °C to 80 °C or further to 70 °C resulted in an obvious decrease of the product yield (from 98% to 91%, and 72%, respectively, Table 1, entries 8, 9 and 10) thus indicating that performing the reaction at 90 °C was essential for high efficiency of the transformation. Additional screening revealed that decreasing the amount of K_2_CO_3_ to 2.0 equiv or 1.0 equiv affected the cyclization process significantly to provide benzo-oxetes in 81% and 59% yield, respectively (Table 1, entries 11 and 12).

**Table 1:** Screening and optimization of the reaction conditions.^a^

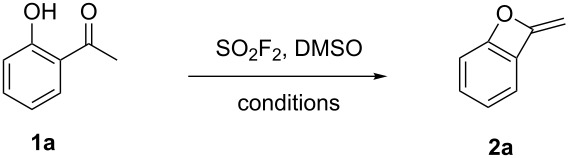				

Entry	Base (equiv)	*T* (°C)	*t* (h)	Yield (**2a**, %)^b^

1	KOAc (3)	90	15	76
2	NaOEt (3)	90	15	50
3	NaOH (3)	90	15	76
4	NaOAc (3)	90	15	50
5	K_2_CO_3_ (3)	90	15	90
6	K_2_CO_3_ (3)	90	5	76
7	K_2_CO_3_ (3)	90	18	94
**8**	**K_2_CO_3_ (3)**	**90**	**20**	**98**
9	K_2_CO_3_ (3)	80	20	91
10	K_2_CO_3_ (3)	70	20	72
11	K_2_CO_3_ (2)	90	20	81
12	K_2_CO_3_ (1)	90	20	59

^a^Reaction conditions: a mixture of 1-(2-hydroxyphenyl)ethanone (**1a**, 1.0 mmol, 1.0 equiv), base (X mmol, X equiv) in DMSO (2.0 mL) was stirred at the indicated temperature, under a SO_2_F_2_ atmosphere (balloon) for 5–20 h. ^b^Isolated yields.

**Scheme 2:**
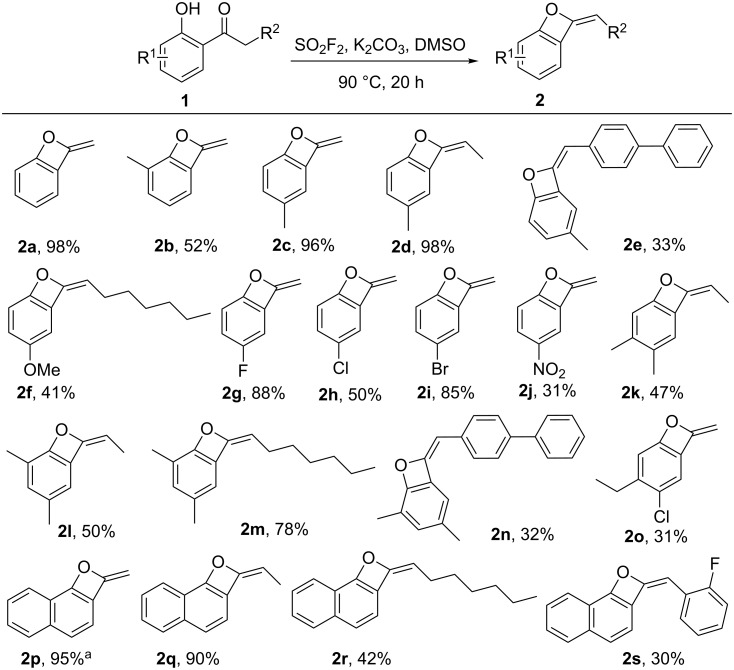
Substrate scope of the SO_2_F_2_-mediated transformation of 2'-hydroxyacetophenones to benzo-oxetes. Reaction conditions: a mixture of 1-(2-hydroxyphenyl)ethanone (**1a**, 1.0 mmol, 1.0 equiv), K_2_CO_3_ (3.0 mmol, 3.0 equiv) in DMSO (2.0 mL) was stirred at 90 ° C under a SO_2_F_2_ atmosphere (balloon) for 20 h. Yields refer to isolated yields. ^a^Reaction performed at 50 °C.

With the optimized reaction conditions in hand, we next evaluated the generality of the SO_2_F_2_-mediated transformation by subjecting diverse 2'-hydroxyacetophenones to the reaction (Scheme 2) [38–39]. Under the optimal reaction conditions, various 2'-hydroxyacetophenones containing both electron-withdrawing and electron-donating groups in the *ortho*, *meta* or *para-*positions of the aryl ring (**1b–j**) efficiently were transformed to the corresponding benzo-oxetes (**2b–j**) in 31– 98% yields. Furthermore, also multifunctionalized 2'-hydroxyacetophenones **1k–o** were converted smoothly to the corresponding benzo-oxetes (**2k–o**) affording the products in 31% to 78% yield. In addition, naphthalenones **1p–s** also successfully were transformed to their corresponding benzo-oxetes **2p–s** in 30–95% yield under identical reaction conditions. For the substituted structure, there is one single isomer obtained, which was proposed to be *E*-configured. However, the exact configuration was not confirmed [40].

**Scheme 3:**
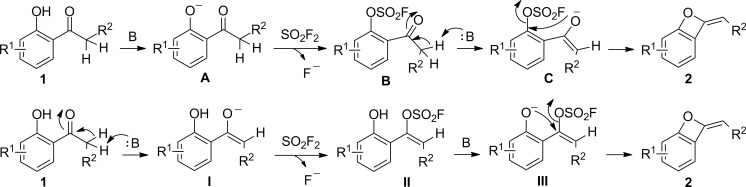
Two proposed reaction mechanisms. B = base.

Two possible reaction mechanisms were proposed for this SO_2_F_2_-mediated transformation of 2'-hydroxyacetophenones to benzo-oxetes (Scheme 3). The first mechanism commences with the deprotonation of 2'-hydroxyacetophenone **1** by the base (K_2_CO_3_) generating phenoxide **A**, which subsequently reacts with SO_2_F_2_ to give intermediate **B** with concomitant release of a fluoride anion. The proton of the acetyl group adjacent to the carbonyl moiety in intermediate **B** is deprotonated by the base (K_2_CO_3_) to generate enolated intermediate **C**, which finally undergoes an intramolecular cyclization furnishing the corresponding benzo-oxete **2**. The second mechanism starts with the elimination of the proton from the acetyl group next to the carbonyl moiety of the 2'-hydroxyacetophenone **1** in the presence of the base to afford enol intermediate **I**. The latter then reacts with SO_2_F_2_ to give intermediate **II** and fluoride anion. The subsequent deprotonation of intermediate **B** by the base generates phenol anion **III**, which finally undergoes an intramolecular cyclization to give the corresponding benzo-oxete **2**.

## Conclusion

We have developed a new protocol for the transformation of 2'-hydroxyacetophenones to benzo-oxetes. This SO_2_F_2_-mediated cascade reaction showed exceptional functional group tolerance and a broad substrate scope which, to the best of our knowledge, is the first example of employing cheap and abundant 2'-hydroxyacetophenones as starting materials for the synthesis of benzo-oxetes.

## Supporting Information

File 1 Conditions optimization, characterization data and copies of NMR spectra.
