# Supplementary material for: SO2F2-mediated transformation of 2'-hydroxyacetophenones to benzo-oxetes
Source: Beilstein J Org Chem. 2019 Apr 25;15:976–80. doi: 10.3762/bjoc.15.95 (PMC6541378; doi:10.3762/bjoc.15.95)

BEILSTEIN JOURNAL OF ORGANIC CHEMISTRY

Supporting Information

for

SO_2F_2-mediated transformation of 2'-hydroxyacetophenones to benzo-oxetes

Revathi Lekkala, Ravindar Lekkala, Balakrishna Moku, K. P. Rakesh and Hua-Li Qin

Beilstein J. Org. Chem. **2019,** *15,* 976–980. doi:10.3762/bjoc.15.95

Conditions optimization, characterization data and copies of NMR spectra

License and Terms: This is a supporting information file under the terms of the Creative Commons Attribution License (http://creativecommons.org/licenses/by/4.0). Please note that the reuse, redistribution and reproduction in particular requires that the authors and source are credited.
The license is subject to the *Beilstein Journal of Organic Chemistry* terms and conditions: (https://www.beilstein-journals.org/bjoc)

Table of contents

1. General consideration S2

2. Screening for optimized reaction conditions S3

3. General procedure.. S5

4. Product characterization... S6

5. NMR spectra S14

1. General considerations

All reactions were carried out in dried glassware. Unless otherwise stated, NMR spectra were recorded in $CDCl_3$ on a 500 MHz (for ^1H), 126 MHz (for ^{13}C) spectrometer. All chemical shifts were reported in ppm relative to TMS (^1H NMR, 0 ppm) as internal standards. All the yields mentioned were isolated. The coupling constants were reported in hertz (Hz). The following abbreviations were used to explain the multiplicities: s = singlet, d = doublet, t = triplet, q = quartet, m = multiplet, br = broad. Melting points measured are uncorrected. Unless otherwise noted, reagents and solvents used in this work were purchased from commercial sources and used as received.

2. Screening for optimized reaction conditions

Table S1: Screening the solvent.[a]

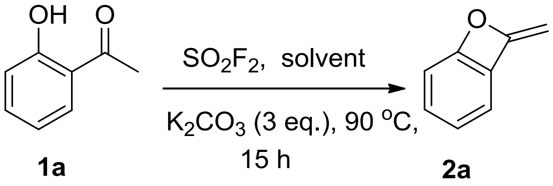

Entry	Solvent	Yield (**2a**, %)[b]
1	DMF	20
**2**	**DMSO**	**90**
3	MeCN	0
4	Acetone	0

[a]Reaction conditions: a mixture of 1-(2-hydroxyphenyl)ethanone (**1a**, 1.0 mmol, 1.0 equiv), K_2CO_3 (3.0 mmol, 3.0 equiv), solvent (2.0 mL) was stirred at 90 °C, charged with a SO_2F_2 balloon for 15 h. [b]Isolated yields.

Table S2. Screening the base.[a]

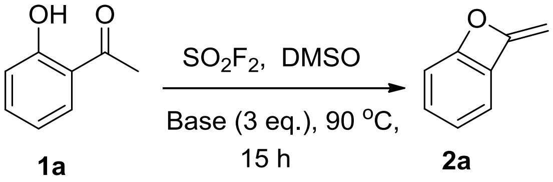

Entry	Base	Yield (**2a**, %)[b]
1	KOAc	76
**2**	**K_2CO_3**	**90**
3	NaOEt	50
4	NaOH	76
5	NaOAc	50

[a]Reaction conditions: a mixture of 1-(2-hydroxyphenyl)ethanone (**1a**, 1.0 mmol, 1.0 equiv), base (3.0 mmol, 3.0 equiv), DMSO (2.0 mL) was stirred at 90 °C, charged with a SO_2F_2 balloon for 15 h. [b]Isolated yields.

Table S3. Screening the reaction time.[a]

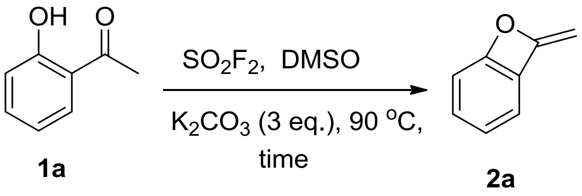

Entry	Time (h)	Yield (**2a,** %)[b]
1	5	76
2	15	90
3	18	94
**4**	**20**	**98**

[a]Reaction conditions: a mixture of 1-(2-hydroxyphenyl)ethanone (**1a**, 1.0 mmol, 1.0 equiv), K_2CO_3 (3.0 mmol, 3.0 equiv), DMSO (2.0 mL) was stirred at 90 °C, charged with a SO_2F_2 balloon for 5–20 h. [b]Isolated yields.

Table S4. Screening the base loading.[a]

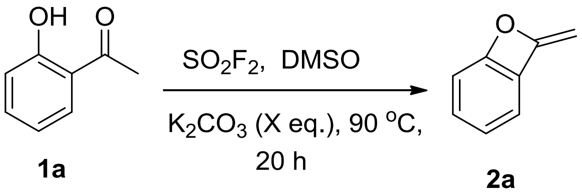

Entry	K_2CO_3 (eq.)	Yield (**2a**, %)[b]
1	1	59
2	1.5	72
3	2	81
4	2.5	89
**5**	**3**	**98**

[a]Reaction conditions: a mixture of 1-(2-hydroxyphenyl)ethanone (**1a**, 1.0 mmol, 1.0 equiv), K_2CO_3 (X mmol, X equiv), DMSO (2.0 mL) was stirred at 90 °C, charged with a SO_2F_2 balloon for 20 h. [b]Isolated yields.

Table S5. Screening the reaction temperature.[a]

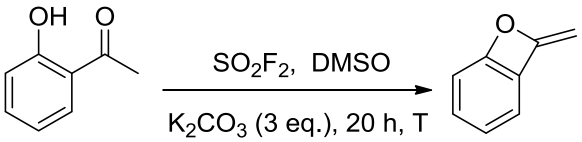

1a **2a**

Entry	*T* (°C)	Yield (**2a**, %)[b]
1	50	60
2	70	72
3	80	91
**4**	**90**	**98**

[a]Reaction conditions: a mixture of 1-(2-hydroxyphenyl)ethanone (**1a**, 1.0 mmol, 1.0 equiv), K_2CO_3 (3.0 mmol, 3.0 equiv), DMSO (2.0 mL) was stirred at the corresponding temperature, charged with a SO_2F_2 balloon for 20 h. [b]Isolated yields.

3. General procedure

General procedure for the synthesis of benzo-oxetes

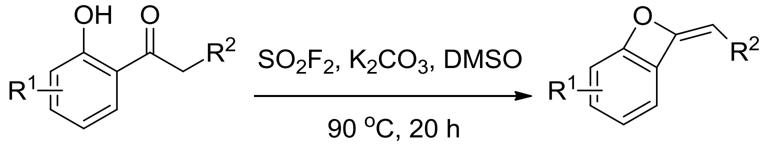

1 **2**

An oven-dried reaction flask (25 mL) containing a stirring bar was charged with 2-hydroxyacetophenone (**1**, 1.0 mmol, 1.0 equib) and K_2CO_3 (3.0 mmol, 3.0 equiv) and the tube was then sealed with a septum. Then DMSO (2.0 mL) was added through a syringe and SO_2F_2 gas (sulfuryl fluoride) was introduced by a needle from a balloon filled with the gas (degassed with SO_2F_2 for 10–30 s). The reaction mixture was vigorously stirred at 90 °C for 20 h. When the 2-hydroxyacetophenone was consumed (monitoring by TLC), the mixture was diluted with water (50 mL) and extracted with EtOAc (3 × 10 mL). The combined organic layer was washed with brine (25 mL), dried over anhydrous Na_2SO_4, and concentrated to dryness. The residue was purified by silica gel chromatography through gradient elution with EtOAc/petroleum ether to afford pure products **2**.

4. Product characterization

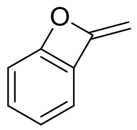

2a

8-Methylene-7-oxabicyclo[4.2.0]octa-1(6),2,4-triene (**2a**). Petroleum ether/ethyl acetate = 10:1 (v/v) as eluent for column chromatography. Yellow liquid (116 mg from 1-(2-hydroxyphenyl)ethanone **1a**, isolated yield 98%). IR (neat): γ_{max}: 3090, 1674, 1580, 882 cm^{-1}; ^{1}H NMR (500 MHz, $CDCl_3$) δ 7.63 (dd, *J* = 8 Hz, *J* = 0.5 Hz, 1H), 7.48-7.45 (m, 1H), 7.31 (t, *J* = 7.5 Hz, 1H), 7.15 (d, *J* = 8.5 Hz, 1H), 5.52 (d, *J* = 4 Hz, 1H), 5.24 (d, *J* = 4 Hz, 1H). ^{13}C NMR (126 MHz, $CDCl_3$) δ 150.7, 148.6, 132.3, 126.6, 125.1, 118.9, 115.8, 99.0. ESI-MS HRMS calculated for C_8H_6O $[M+H]^+$ 119.0497, found. 119.0494. Rf = 0.3 (10:1, PE/EA).

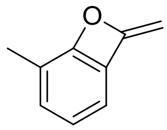

2b

5-Methyl-8-methylene-7-oxabicyclo[4.2.0]octa-1(6),2,4-triene (**2b**). Petroleum ether/ethyl acetate = 15:1 (v/v) as eluent for column chromatography. Red liquid (69 mg from 1-(2-hydroxy-3-methylphenyl)ethanone **1b**, isolated yield 52%). IR (neat): γ_{max}: 3051, 2941, 1643, 1593, 863 cm^{-1}; ^{1}H NMR (500 MHz, $CDCl_3$) δ 7.45 (dd, *J* = 8 Hz, *J* = 1 Hz, 1H), 7.30 (d, *J* = 7.5 Hz, 1H), 7.19-7.16 (m, 1H), 5.48 (d, *J* = 4 Hz, 1H), 5.21 (d, *J* = 4 Hz, 1H), 2.33 (s, 3H). ^{13}C NMR (126 MHz, $CDCl_3$) δ 150.9, 147.3, 133.6, 128.5, 126.0, 122.7, 115.5, 98.8, 15.3. ESI-MS HRMS calculated for C_9H_8O $[M+H]^+$ 133.0653, found. 133.0655. Rf = 0.4 (10:1, PE/EA).

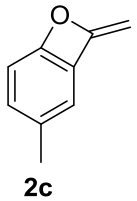

2c

3-Methyl-8-methylene-7-oxabicyclo[4.2.0]octa-1(6),2,4-triene (**2c**). Petroleum ether/ethyl acetate = 15:1 (v/v) as eluent for column chromatography. Reddish black liquid (127 mg from 1-(2-hydroxy-5-methylphenyl)ethanone **1c**, isolated yield 96%). IR (neat): γ_{max}: 3083, 2831, 1655, 1583, 844 cm^{-1};^{1}H NMR (500 MHz, $CDCl_3$) δ 7.32 (d, *J* = 1 Hz, 1H), 7.17 (dd, *J* = 9 Hz, *J* = 2 Hz, 1H), 6.95 (d, *J* = 8.5 Hz, 1H), 5.40 (d, *J* = 4 Hz, 1H), 5.12 (d, *J* = 4 Hz, 1H), 2.30 (s, 3H). ^{13}C NMR (126 MHz, $CDCl_3$) δ 149.3, 147.5, 135.1, 127.8, 120.5, 119.5,

117.5, 100.5, 27.7. ESI-MS HRMS calculated for C_9H_8O [M+H]$^+$ 133.0653, found. 133.0658. Rf = 0.4 (10:1, PE/EA).

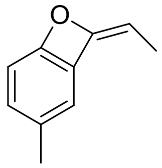

2d

(*E*)-8-Ethylidene-3-methyl-7-oxabicyclo[4.2.0]octa-1(6),2,4-triene (**2d**). Petroleum ether/ ethyl acetate = 30:1 (v/v) as eluent for column chromatography. Yellow solid (143 mg from 1-(2-hydroxy-5-methylphenyl)propan-1-one **1d**, isolated yield 98%). M.p. 87-89 °C. IR (neat): γ_{max}: 3072, 2951, 1637, 1583, 794 cm^{-1}; ^{1}H NMR (500 MHz, $CDCl_3$) δ 7.23 (d, *J* = 1 Hz, 1H), 7.09 (dd, *J* = 8.5 Hz, *J* = 1.5 Hz, 1H), 6.91 (d, *J* = 8.5 Hz, 1H), 5.88 (q, *J* = 7 Hz, 1H), 2.27 (s, 3H), 1.83 (d, *J* = 7 Hz, 3H). ^{13}C NMR (126 MHz, $CDCl_3$) δ 145.9, 144.9, 136.3, 131.7, 123.9, 118.3, 116.1, 110.5, 21.0, 10.3. ESI-MS HRMS calculated for $C_{10}H_{10}O$ [M+H]$^+$ 147.0810, found. 147.0814. Rf = 0.5 (20:1, PE/EA).

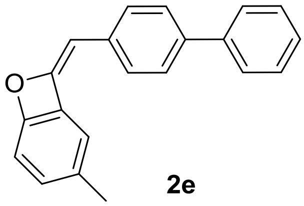

2e

(*E*)-8-([1,1'-Biphenyl]-4-ylmethylene)-3-methyl-7-oxabicyclo[4.2.0]octa-1(6),2,4-triene (**2e**). Petroleum ether/ethyl acetate = 40:1 (v/v) as eluent for column chromatography. White solid (93 mg from 2-([1,1'-biphenyl]-4-yl)-1-(2-hydroxy-5-methylphenyl)ethanone **1e**, isolated yield 33%). M.p. 77-79 °C. IR (neat): γ_{max}: 3064, 2880, 1673, 1581, 849 cm^{-1}; ^{1}H NMR (500 MHz, $CDCl_3$) δ 7.65-7.61 (m, 7H), 7.48-7.45 (m, 3H), 7.39-7.37 (m, 1H), 7.30-7.28 (m, 1H), 7.22 (dd, *J* = 8.5 Hz, *J* = 1.5 Hz, 1H), 2.40 (s, 3H). ^{13}C NMR (126 MHz, $CDCl_3$) δ 148.0, 142.0, 140.4, 138.9, 134.2, 132.4, 130.6, 129.0, 127.9, 127.3, 127.2, 121.3, 117.7, 96.1, 83.0, 20.9. ESI-MS HRMS calculated for $C_{21}H_{16}O$ [M+H]$^+$ 285.1279, found. 285.1275. Rf = 0.6 (20:1, PE/EA).

Note: In the ^{13}C NMR spectrum of **2e**, theoretically, there should be seventeen peaks. Due to the compact overlaying, it is difficult to specify the overlaying peaks.

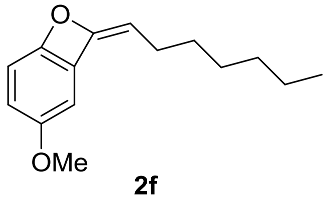

2f

(*E*)-8-Heptylidene-3-methoxy-7-oxabicyclo[4.2.0]octa-1(6),2,4-triene (**2f**). Petroleum ether/ ethyl acetate = 15:1 (v/v) as eluent for column chromatography. Yellow liquid (95 mg from 1-(2-hydroxy-5-methoxyphenyl)octan-1-one **1f**, isolated yield 41%). IR (neat): γ_{max}: 3041, 2930, 1642, 1593, 849 cm^{-1}; ^{1}H NMR (500 MHz, $CDCl_3$) δ 7.03 (d, *J* = 9 Hz, 1H), 6.97 (d, *J* = 2.5 Hz, 1H), 6.91 (dd, *J* = 9.5 Hz, *J* = 3 Hz, 1H), 5.89 (t, *J* = 8 Hz, 1H), 3.82 (s, 3H), 2.36 (q, *J* = 7.5 Hz, 2H), 1.52-1.47 (m, 2H), 1.39-1.29 (m, 6H), 0.90 (t, *J* = 7 Hz, 3H). ^{13}C NMR (126 MHz, $CDCl_3$) δ 157.5, 144.0, 142.0, 119.7, 117.2, 117.0, 116.5, 108.0, 56.0, 31.7, 29.0, 24.8, 22.7, 14.2. ESI-MS HRMS calculated for $C_{15}H_{20}O_2$ $[M+H]^+$ 233.1542, found. 233.1546. Rf = 0.4 (10:1, PE/EA).

Note: In the ^{13}C NMR spectrum of **2f**, theoretically, there should be fifteen peaks. Due to the compact overlaying, it is difficult to specify the overlaying peaks.

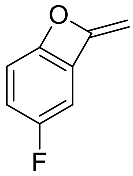

2g

3-Fluoro-8-methylene-7-oxabicyclo[4.2.0]octa-1(6),2,4-triene (**2g**). Petroleum ether/ethyl acetate = 15:1 (v/v) as eluent for column chromatography. Yellow solid (120 mg from 1-(5-fluoro-2-hydroxyphenyl)ethanone **1g**, isolated yield 88%). M.p. 77-79 ^{o}C. IR (neat): γ_{max}: 3081, 1653, 1583, 884 cm^{-1}; ^{1}H NMR (500 MHz, $CDCl_3$) δ 7.30 (dd, *J* = 8.5 Hz, *J* = 2.5 Hz, 1H), 7.20-7.13 (m, 2H), 5.49 (d, *J* = 4 Hz, 1H), 5.31 (d, *J* = 4.5 Hz, 1H). ^{13}C NMR (126 MHz, $CDCl_3$) δ 160.1 (d, *J* = 247.0 Hz), 149.7 (d, *J* = 3.15 Hz), 144.6 (d, *J* = 2.5 Hz), 120.7 (d, *J* = 8.6 Hz), 119.5 (d, *J* = 24.4 Hz), 117.2 (d, *J* = 8.6 Hz), 111.5 (d, *J* = 26.5 Hz), 100.3. ESI-MS HRMS calculated for C_8H_5OF $[M+H]^+$ 137.0403, found. 137.0405. Rf = 0.4 (10:1, PE/EA).

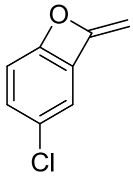

2h

3-Chloro-8-methylene-7-oxabicyclo[4.2.0]octa-1(6),2,4-triene (**2h**). Petroleum ether/ethyl acetate = 15:1 (v/v) as eluent for column chromatography. Yellow liquid (76 mg from 1-(5-chloro-2-hydroxyphenyl)ethanone **1h**, isolated yield 50%). IR (neat): γ_{max}: 3061, 1673, 1578, 836 cm^{-1}; ^{1}H NMR (500 MHz, $CDCl_3$) δ 7.63 (d, *J* = 2.5 Hz, 1H), 7.56 (d, *J* = 2.5 Hz, 1H), 7.41 (d, *J* = 8.5 Hz, 1H), 7.25-7.23 (m, 1H), 6.71 (dd, *J* = 2 Hz, *J* = 0.5 Hz, 1H). ^{13}C NMR

(126 MHz, $CDCl_3$) δ 153.3, 146.4, 129.0, 128.5, 124.7, 120.9, 112.5, 106.4. ESI-MS HRMS calculated for C_8H_5OCl $[M+H]^+$ 153.0107, found. 153.0104. Rf = 0.4 (10:1, PE/EA).

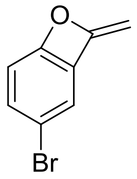

2i

3-Bromo-8-methylene-7-oxabicyclo[4.2.0]octa-1(6),2,4-triene (**2i**). Petroleum ether/ethyl acetate = 20:1 (v/v) as eluent for column chromatography. Yellow solid (165 mg from 1-(5-bromo-2-hydroxyphenyl)ethanone **1i**, isolated yield 85%). M.p. 99-101 °C. IR (neat): γ_{max}: 3052, 1635, 1588, 783 cm^{-1}; ^1H NMR (500 MHz, $CDCl_3$) δ 7.74 (d, *J* = 2.5 Hz, 1H), 7.56 (dd, *J* = 9 Hz, *J* = 2.5 Hz, 1H), 7.04 (d, *J* = 9 Hz, 1H), 5.53 (d, *J* = 4.5 Hz, 1H), 5.31 (d, *J* = 4.5 Hz, 1H). ^{13}C NMR (126 MHz, $CDCl_3$) δ 149.4, 147.6, 135.1, 127.8, 120.5, 119.5, 117.6, 100.4. ESI-MS HRMS calculated for C_8H_5OBr $[M+H]^+$ 196.9602, found. 196.9606. Rf = 0.5 (10:1, PE/EA).

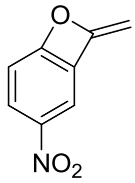

2j

8-Methylene-3-nitro-7-oxabicyclo[4.2.0]octa-1(6),2,4-triene (**2j**). Petroleum ether/ethyl acetate = 10:1 (v/v) as eluent for column chromatography. White solid (51 mg from 1-(2-hydroxy-5-nitrophenyl)ethanone **1j**, isolated yield 31%). M.p. 107-108 °C. IR (neat): γ_{max}: 3095, 1667, 1581, 881 cm^{-1}; ^1H NMR (500 MHz, $CDCl_3$) δ 8.55 (d, *J* = 2.5 Hz, 1H), 8.24 (dd, *J* = 9 Hz, *J* = 2 Hz, 1H), 7.79 (d, *J* = 2.5 Hz, 1H), 7.59 (d, *J* = 9 Hz, 1H), 6.93 (dd, *J* = 2 Hz, *J* = 0.5 Hz, 1H). ^{13}C NMR (126 MHz, $CDCl_3$) δ 157.9, 148.1, 144.5, 128.0, 120.4, 118.0, 111.9, 107.8. ESI-MS HRMS calculated for $C_8H_5O_3N$ $[M+H]^+$ 164.0348, found. 164.0345. Rf = 0.4 (5:1, PE/EA).

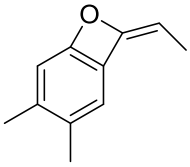

2k

(E)-8-Ethylidene-3,4-dimethyl-7-oxabicyclo[4.2.0]octa-1(6),2,4-triene (**2k**). Petroleum ether/ ethyl acetate = 30:1 (v/v) as eluent for column chromatography. Yellow liquid (75 mg from 1-(2-hydroxy-4,5-dimethylphenyl)propan-1-one **1k**, isolated yield 47%). IR (neat): γ_{max}: 3032, 2873, 1654, 1580, 876 cm^{-1}; ^{1}H NMR (500 MHz, $CDCl_3$) δ 7.14 (s, 1H), 7.02 (s, 1H), 5.93 (q, J = 7 Hz, 1H), 2.31 (s, 3H), 2.26 (s, 3H), 1.90 (d, J = 7 Hz, 3H). ^{13}C NMR (126 MHz, $CDCl_3$) δ 145.2, 144.7, 135.5, 133.2, 127.9, 121.6, 116.0, 110.2, 20.9, 15.2, 10.3. ESI-MS HRMS calculated for $C_{11}H_{12}O$ $[M+H]^+$ 161.0966, found. 161.0969. Rf = 0.4 (20:1, PE/EA).

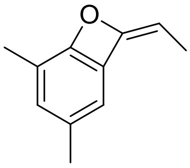

2l

(E)-8-Ethylidene-3,5-dimethyl-7-oxabicyclo[4.2.0]octa-1(6),2,4-triene (**2l**). Petroleum ether/ ethyl acetate = 30:1 (v/v) as eluent for column chromatography. Yellow liquid (80 mg from 1-(2-hydroxy-3,5-dimethylphenyl)propan-1-one **1l**, isolated yield 50%). IR (neat): γ_{max}: 3056, 2876, 1641, 1590, 870 cm^{-1}; ^{1}H NMR (500 MHz, $CDCl_3$) δ 7.14 (s, 1H), 7.02 (s, 1H), 5.93 (q, J = 7 Hz, 1H), 2.30 (s, 3H), 2.25 (s, 3H), 1.90 (d, J = 7 Hz, 3H). ^{13}C NMR (126 MHz, $CDCl_3$) δ 145.1, 144.6, 135.6, 133.2, 127.8, 121.5, 115.9, 110.2, 77.4, 77.2, 76.9, 20.9, 15.2, 10.3. ESI-MS HRMS calculated for $C_{11}H_{12}O$ $[M+H]^+$ 161.0966, found. 161.0969. Rf = 0.4 (20:1, PE/EA).

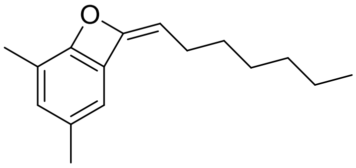

2m

(E)-8-Heptylidene-3,5-dimethyl-7-oxabicyclo[4.2.0]octa-1(6),2,4-triene (**2m**). Petroleum ether/ethyl acetate = 40:1 (v/v) as eluent for column chromatography. Yellow liquid (179 mg from 1-(2-hydroxy-3,5-dimethylphenyl)octan-1-one **1m**, isolated yield 78%). IR (neat): γ_{max}:

3043, 2901, 1610, 1590, 830 cm^{-1}; ^{1}H NMR (500 MHz, $CDCl_3$) δ 7.15 (s, 1H), 7.02 (s, 1H), 5.88 (t, *J* = 7.5 Hz, 1H), 2.37-2.34 (m, 2H), 2.31 (s, 3H), 2.26 (s, 3H), 1.51-1.46 (m, 2H), 1.37-1.31 (m, 6H), 0.90 (s, 3H). ^{13}C NMR (126 MHz, $CDCl_3$) δ 144.8, 144.4, 135.5, 133.2, 127.9, 121.6, 115.9, 115.8, 31.7, 29.1, 29.0, 24.8, 22.7, 21.0, 15.3, 14.2. ESI-MS HRMS calculated for $C_{16}H_{22}O$ $[M+H]^+$ 231.1749, found. 231.1745. Rf = 0.6 (20:1, PE/EA).

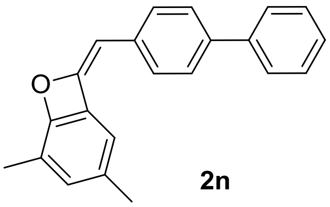

2n

(*E*)-8-([1,1'-Biphenyl]-4-ylmethylene)-3,5-dimethyl-7-oxabicyclo[4.2.0]octa-1(6),2,4-triene (**2n**). Petroleum ether/ethyl acetate = 40:1 (v/v) as eluent for column chromatography. White solid (95 mg from 2-([1,1'-biphenyl]-4-yl)-1-(2-hydroxy-3,5-dimethylphenyl)ethanone **1n**, isolated yield 32%). M.p. 153-154 °C. IR (neat): γ_{max}: 3090, 2921, 1650, 1593, 807 cm^{-1}; ^{1}H NMR (500 MHz, $CDCl_3$) δ 7.84 (d, *J* = 8.5 Hz, 2H), 7.60-7.55 (m, 4H), 7.38 (t, *J* = 7.5 Hz, 2H), 7.30-7.27 (m, 1H), 7.11 (s, 1H), 6.88 (s, 1H), 6.83 (s, 1H), 2.48 (s, 3H), 2.33 (s, 3H). ^{13}C NMR (126 MHz, $CDCl_3$) δ 155.5, 152.6, 141.1, 140.7, 132.5, 129.9, 129.0, 129.0, 127.6, 127.5, 127.1, 126.8, 125.3, 121.0, 118.3, 101.6, 21.4, 15.2. ESI-MS HRMS calculated for $C_{22}H_{18}O$ $[M+H]^+$ 299.1436, found. 299.1432. Rf = 0.6 (20:1, PE/EA).

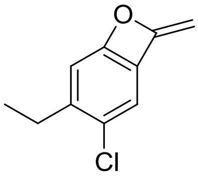

2o

3-Chloro-4-ethyl-8-methylene-7-oxabicyclo[4.2.0]octa-1(6),2,4-triene (**2o**). Petroleum ether/ ethyl acetate = 30:1 (v/v) as eluent for column chromatography. Yellow liquid (56 mg from 1-(4-ethyl-2-hydroxy-5-methylphenyl)ethanone **1o**, isolated yield 31%). IR (neat): γ_{max}: 3031, 2843, 1655, 1580, 787 cm^{-1}; ^{1}H NMR (500 MHz, $CDCl_3$) δ 7.58-7.57 (m, 2H), 7.38 (s, 1H), 6.68 (dd, *J* = 2 Hz, *J* = 1 Hz, 1H), 2.85 (q, *J* = 7.5 Hz, 2H), 1.28 (t, *J* = 7.5 Hz, 3H). ^{13}C NMR (126 MHz, $CDCl_3$) δ 154.1, 145.7, 138.0, 128.6, 126.6, 121.3, 111.8, 106.1, 27.4, 14.4. ESI-MS HRMS calculated for $C_{10}H_9OCl$ $[M+H]^+$ 181.0420, found. 181.0424. Rf = 0.4 (20:1, PE/EA).

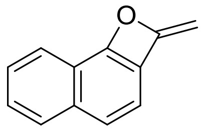

2p

2-Methylene-2H-naphtho[1,2-*b*]oxete (**2p**). Petroleum ether/ethyl acetate = 20:1 (v/v) as eluent for column chromatography. Brown solid (160 mg from 1-(1-hydroxynaphthalen-2-yl)ethanone **2p**, reaction was performed at 50 °C (instead of 90 °C), isolated yield 95%). M.p. 87-89 °C. IR (neat): γ_{max}: 3045, 1659, 1589, 874 cm^{-1}; ^{1}H NMR (500 MHz, $CDCl_3$) δ 8.19-8.17 (m, 1H), 7.88-7.85 (m, 1H), 7.74 (d, *J* = 8.5 Hz, 1H), 7.66-7.63 (m, 2H), 7.56 (d, *J* = 8.5 Hz, 1H), 5.58 (d, *J* = 4 Hz, 1H), 5.32 (d, *J* = 4 Hz, 1H). ^{13}C NMR (126 MHz, $CDCl_3$) δ 151.1, 144.7, 135.1, 128.9, 128.2, 128.0, 126.4, 123.9, 121.4, 120.2, 111.0, 99.5. ESI-MS HRMS calculated for $C_{12}H_8O$ [M+H]$^+$ 169.0653, found. 169.0656. Rf = 0.3 (20:1, PE/EA).

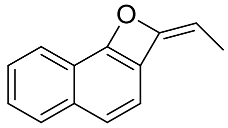

2q

(*E*)-2-Ethylidene-2H-naphtho[1,2-*b*]oxete (**2q**). Petroleum ether/ethyl acetate = 30:1 (v / v) as eluent for column chromatography. Yellow solid (164 mg from 1-(1-hydroxynaphthalen-2-yl)propan-1-one **1q**, isolated yield 90%). M.p. 142-144 °C. IR (neat): γ_{max}: 3075, 2961, 1647, 1582, 854 cm^{-1}; ^{1}H NMR (500 MHz, $CDCl_3$) δ 8.14 (d, *J* = 8 Hz, 1H), 7.84 (d, *J* = 8 Hz, 1H), 7.69 (d, *J* = 8.5 Hz, 1H), 7.64-7.58 (m, 2H), 7.49 (d, *J* = 9 Hz, 1H), 6.06 (q, *J* = 7 Hz, 1H), 1.99 (d, *J* = 7 Hz, 3H). ^{13}C NMR (126 MHz, $CDCl_3$) δ 148.5, 134.1, 129.1, 128.0, 128.0, 127.5, 127.4, 127.3, 122.2, 116.2, 94.6, 75.0, 4.8. ESI-MS HRMS calculated for $C_{13}H_{10}O$ [M+H]$^+$ 183.0810, found. 183.0814. Rf = 0.5 (20:1, PE/EA).

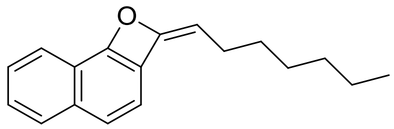

2r

(*E*)-2-Heptylidene-2H-naphtho[1,2-*b*]oxete (**2r**). Petroleum ether/ethyl acetate = 30:1 (v/v) as eluent for column chromatography. Yellow liquid (106 mg from 1-(1-hydroxynaphthalen-2-yl)octan-1-one **1r**, isolated yield 42%). IR (neat): γ_{max}: 3052, 2930, 1678, 1593, 756 cm^{-1}; ^{1}H NMR (500 MHz, $CDCl_3$) δ 8.03 (d, *J* = 8.5 Hz, 1H), 7.86 (d, *J* = 8 Hz, 1H), 7.78 (d, *J* = 8.5 Hz, 1H), 7.64 (t, *J* = 7.5 Hz, 1H), 7.57 (t, *J* = 7.5 Hz, 1H), 7.50 (d, *J* = 8.5 Hz, 1H), 2.53 (t, *J* = 7.5 Hz, 2H), 1.71-1.65 (m, 2H), 1.56-1.49 (m, 3H), 1.35-1.27 (m, 4H), 0.92 (t, *J* = 6.5 Hz,

3H). ^{13}C NMR (126 MHz, $CDCl_3$) δ 147.1, 134.0, 129.1, 128.4, 128.4, 128.2, 127.6, 126.3, 120.9, 116.0, 100.0, 74.6, 31.5, 28.8, 28.5, 22.7, 19.9, 14.2. ESI-MS HRMS calculated for $C_{18}H_{20}O$ $[M+H]^+$ 253.1592, found. 253.1595. Rf = 0.4 (20:1, PE/EA).

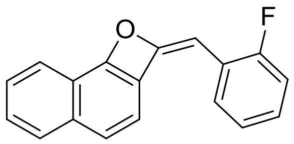

2s

(*E*)-2-(2-Fluorobenzylidene)-2*H*-naphtho[1,2-*b*]oxete (**2s**). Petroleum ether/ethyl acetate = 30:1 (v/v) as eluent for column chromatography. White solid (79 mg from 2-(2-fluorophenyl)-1-(1-hydroxynaphthalen-2-yl)ethanone **1s**, isolated yield 30%). M.p. 99-100 ^{o}C. IR (neat): γ_{max}: 3051, 2936, 1660, 1590, 835 cm^{-1}; ^{1}H NMR (500 MHz, $CDCl_3$) δ 8.41 (d, *J* = 8 Hz, 1H), 8.18 (t, *J* = 7.5 Hz, 1H), 7.95 (d, *J* = 8 Hz, 1H), 7.68 (s, 2H), 7.62 (t, *J* = 7.5 Hz, 1H), 7.51 (t, *J* = 7.5 Hz, 1H), 7.37 (s, 1H), 7.32-7.29 (m, 2H), 7.22-7.19 (m, 1H). ^{13}C NMR (126 MHz, $CDCl_3$) δ 159.445 (d, *J* = 252.1 Hz), 149.9, 149.4 (d, *J* = 3.4 Hz), 131.9, 129.3 (d, *J* = 8.4 Hz), 128.6, 126.9 (d, *J* = 2.9 Hz), 126.5, 125.4, 125.1 (d, *J* = 1.5 Hz), 124.6 (d, *J* = 3.5 Hz), 123.9, 121.4, 120.3, 119.9, 119.2 (d, *J* = 11.7 Hz), 116.3 (d, *J* = 21.5 Hz), 107.8 (d, *J* = 12.6 Hz). ESI-MS HRMS calculated for $C_{18}H_{11}OF$ $[M+H]^+$ 263.0872, found. 263.0875. Rf = 0.5 (20:1, PE/EA).

5. NMR spectra

2a, ^{1}H NMR, 500 MHz, $CDCl_3$

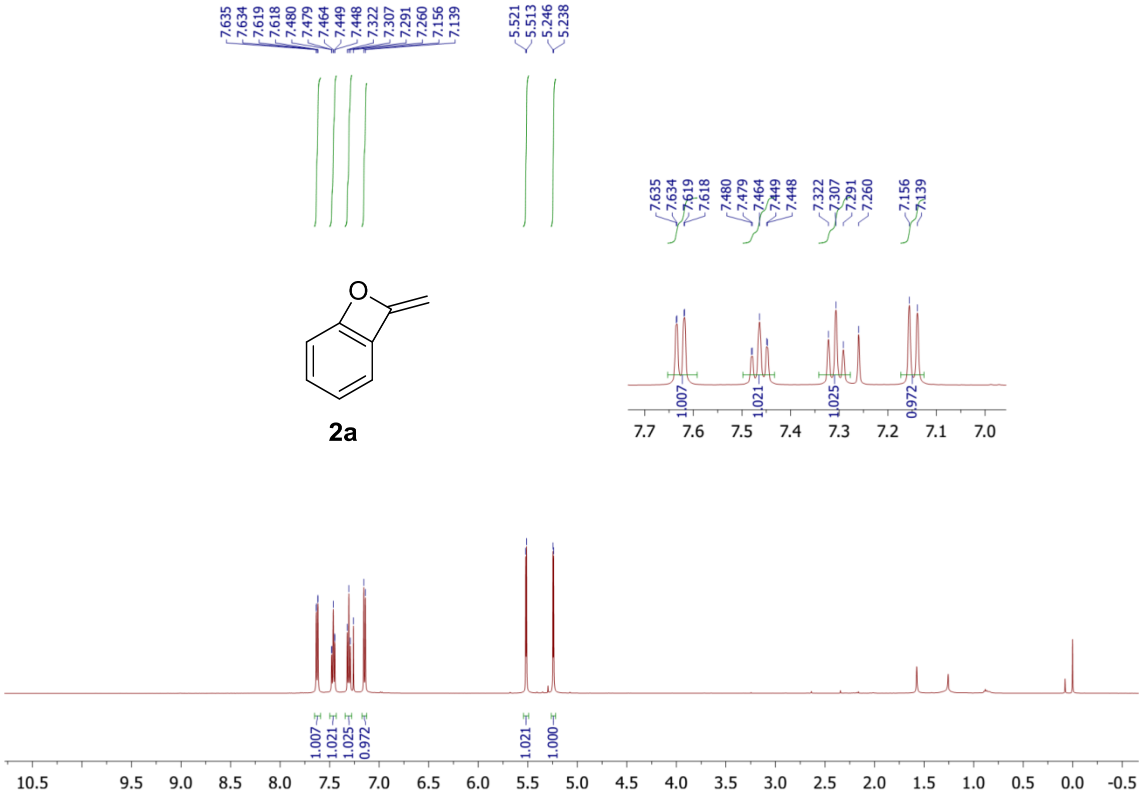

2a, ^{13}C NMR, 126 MHz, $CDCl_3$

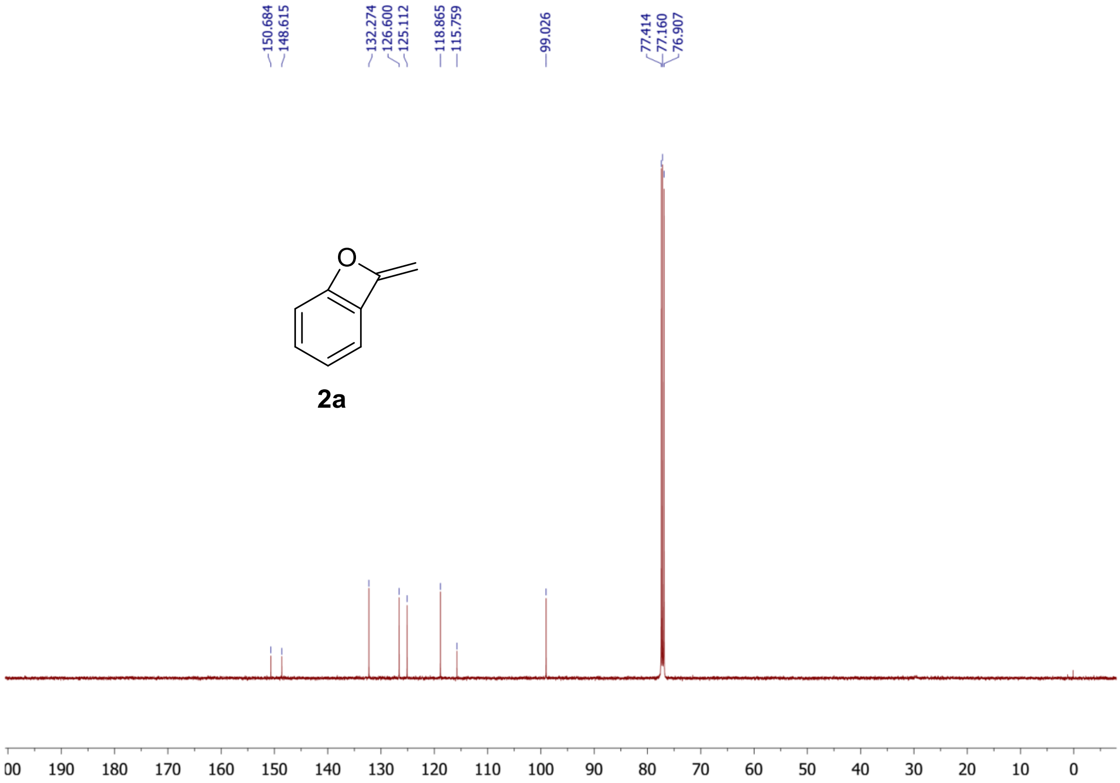

2b, ^{1}H NMR, 500 MHz, $CDCl_3$

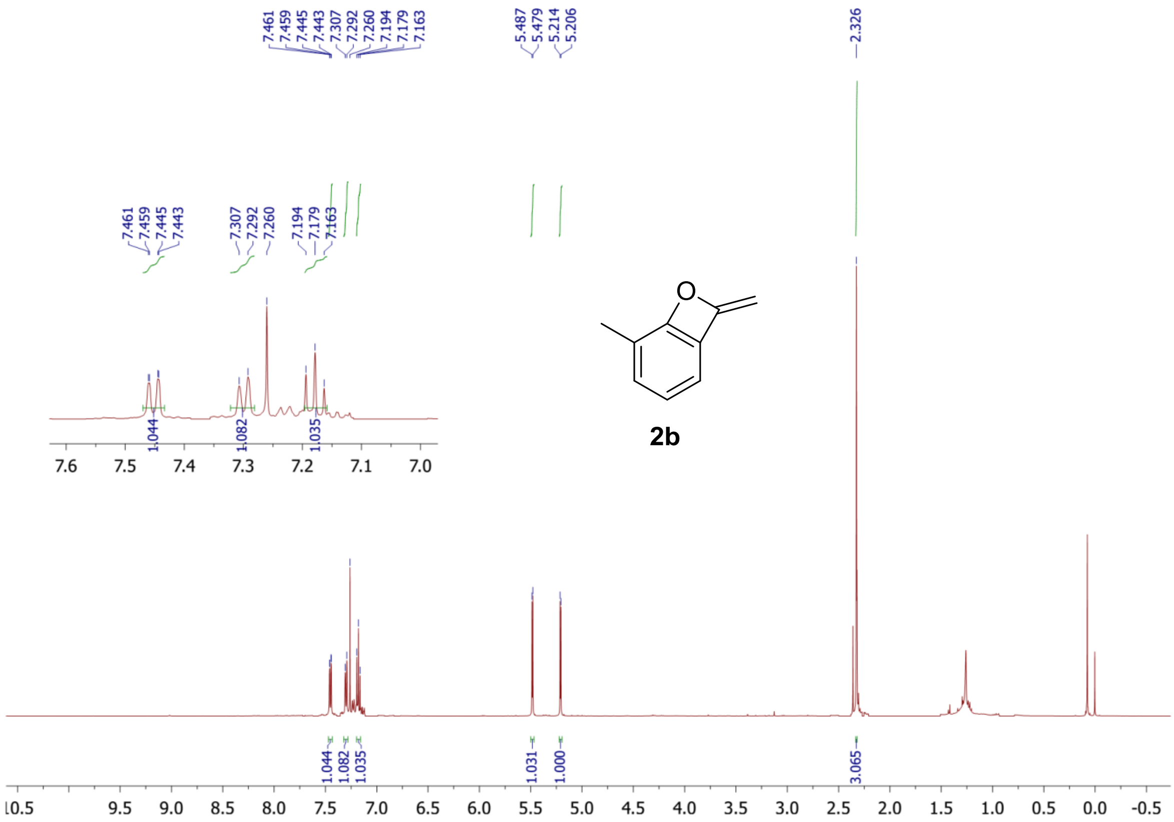

2b, ^{13}C NMR, 126 MHz, $CDCl_3$

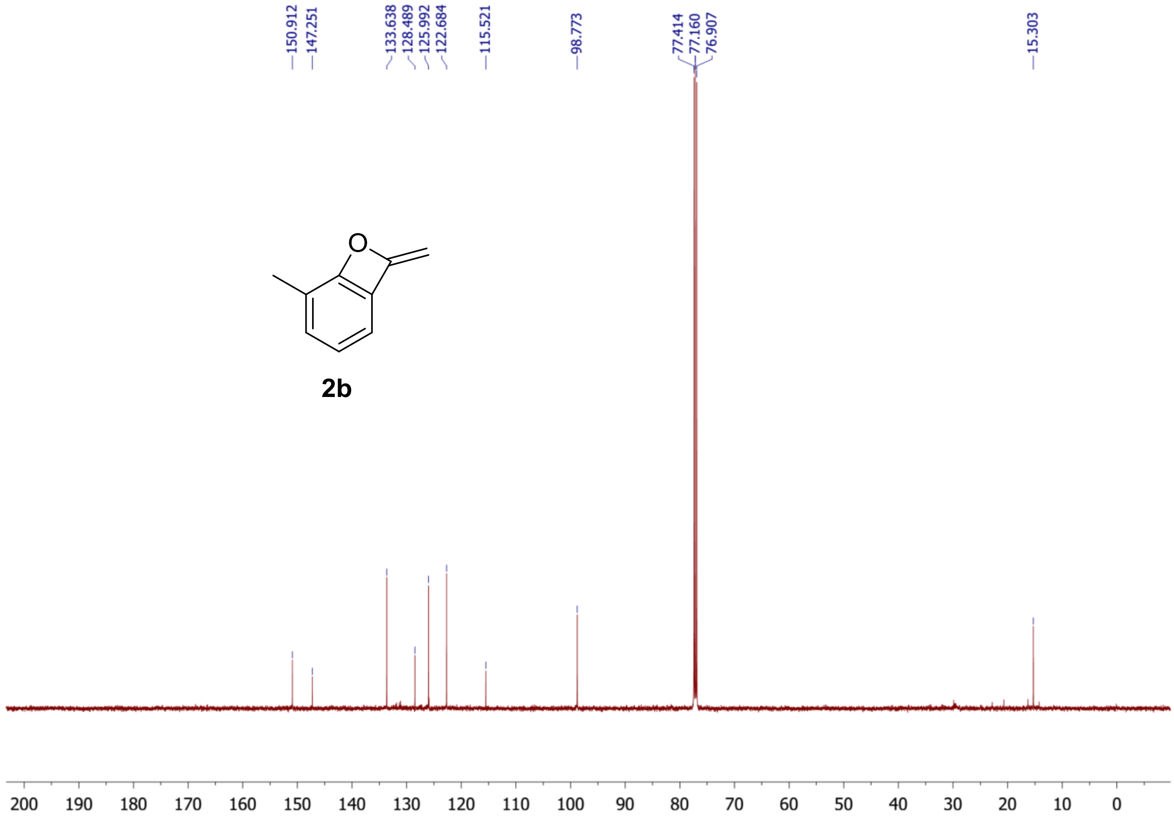

2c, ^{1}H NMR, 500 MHz, $CDCl_3$

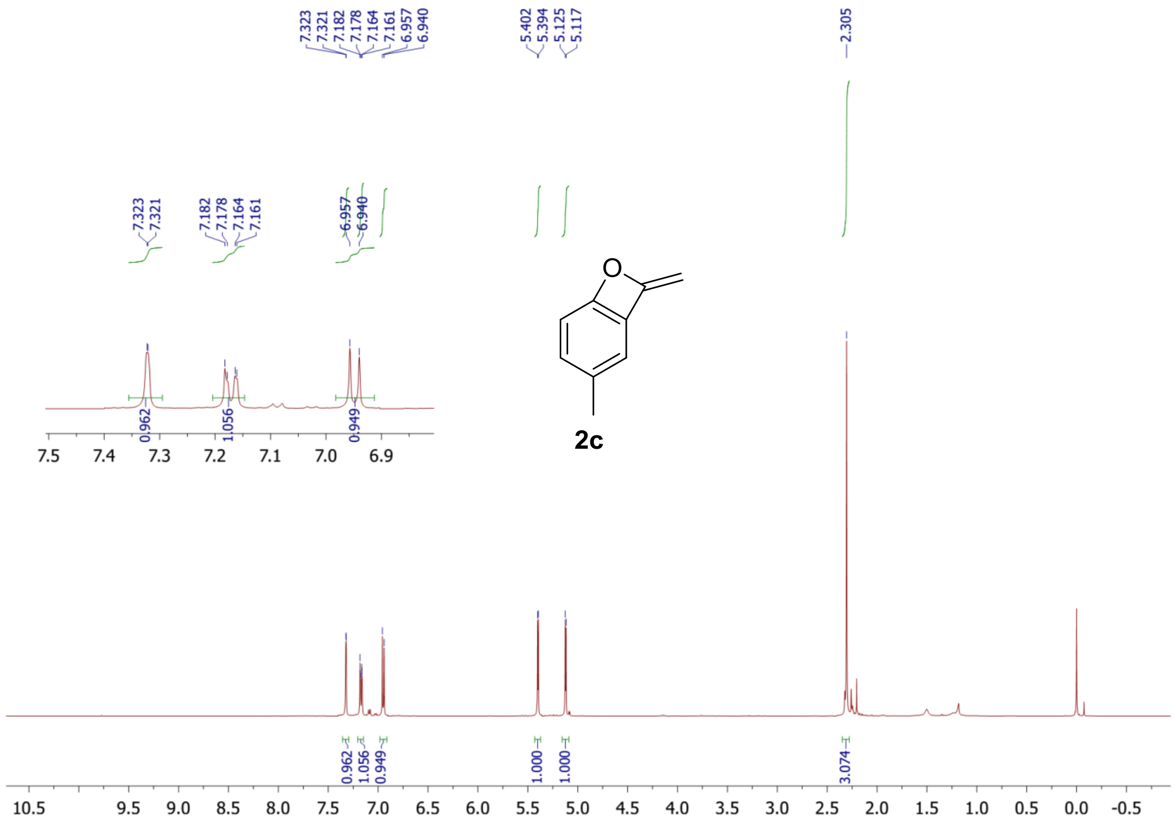

2c, ^{13}C NMR, 126 MHz, $CDCl_3$

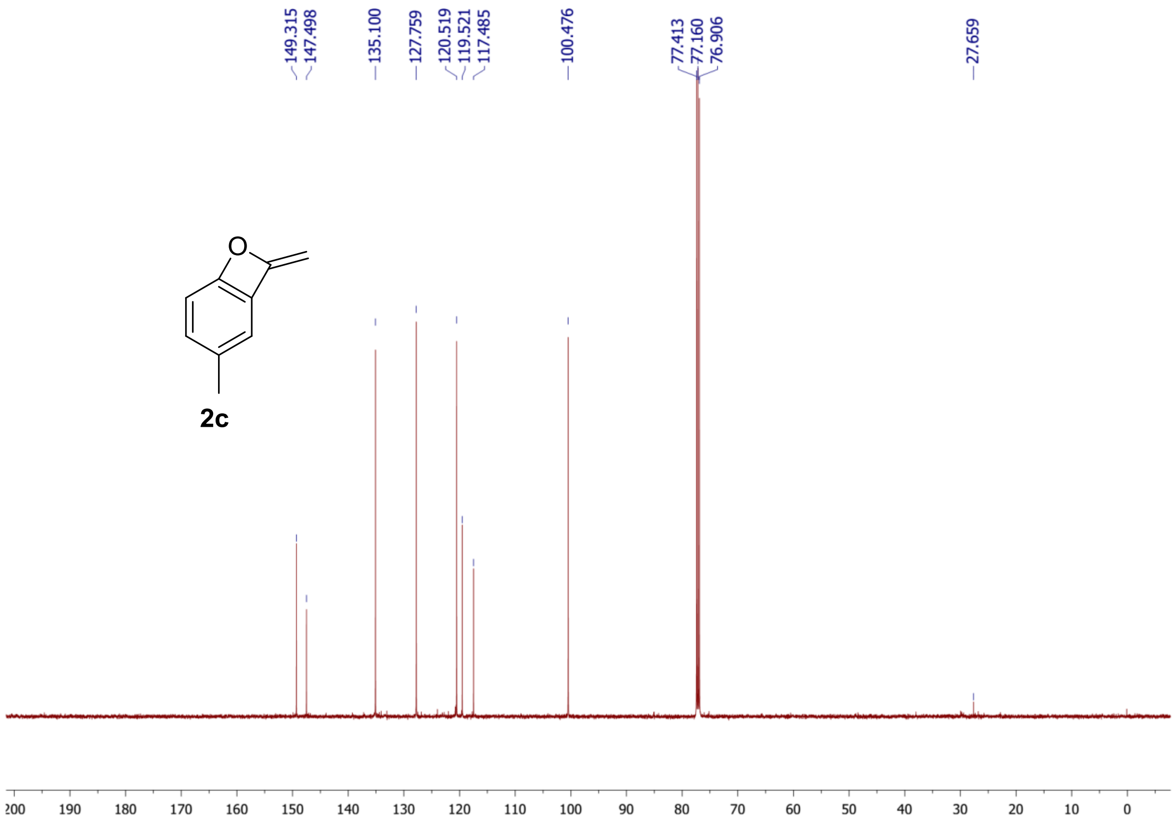

2d, ^{1}H NMR, 500 MHz, $CDCl_3$

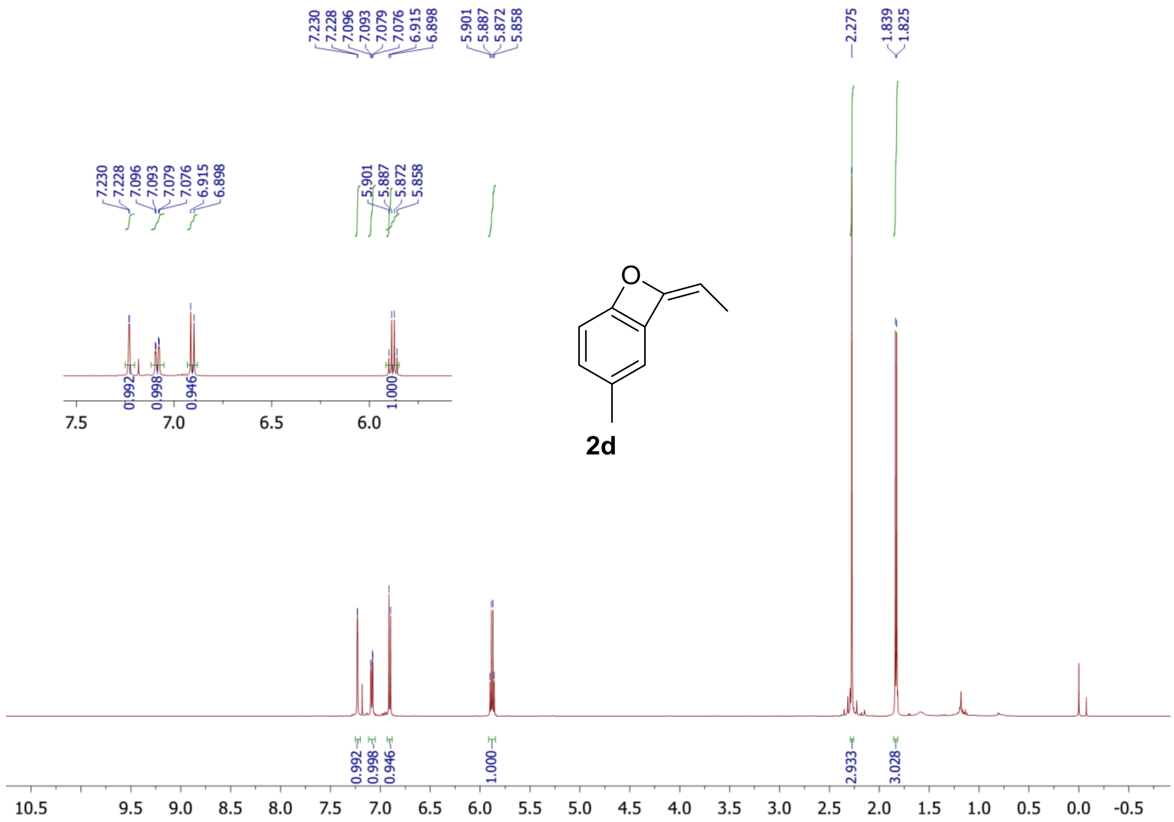

2d, ^{13}C NMR, 126 MHz, $CDCl_3$

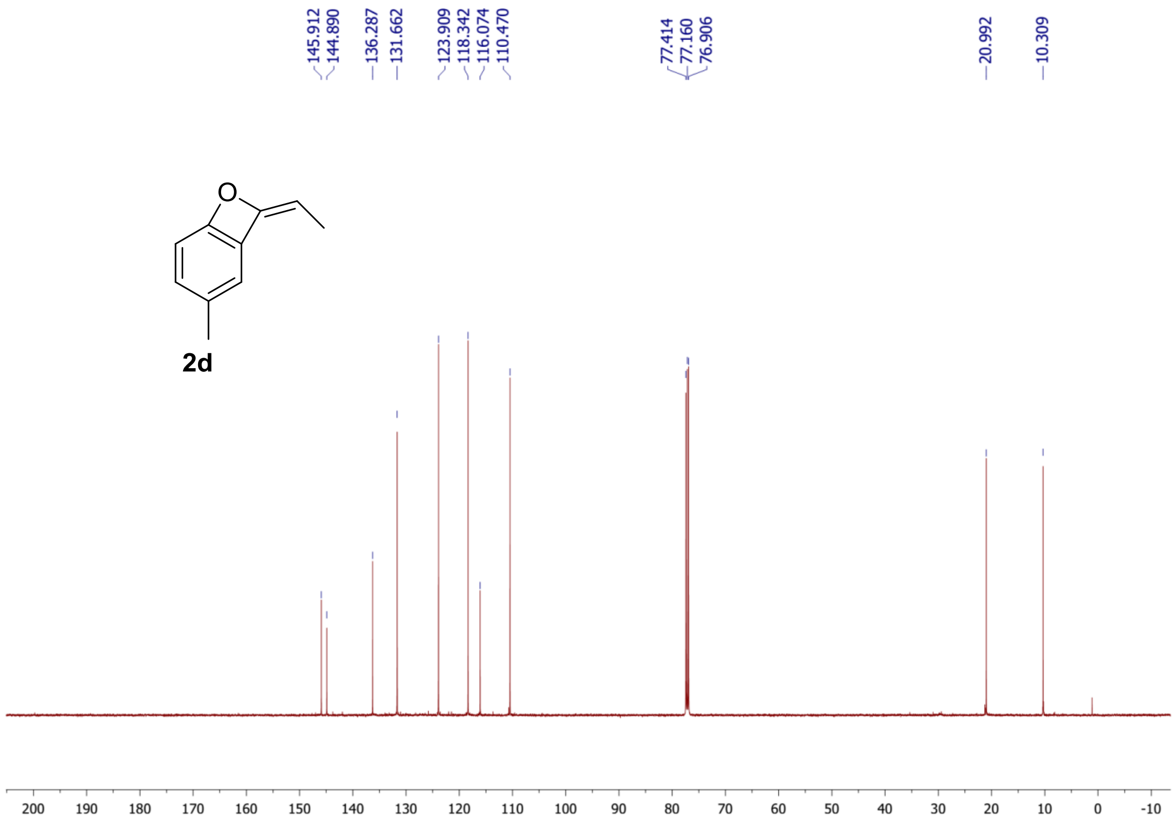

2e, ^{1}H NMR, 500 MHz, $CDCl_3$

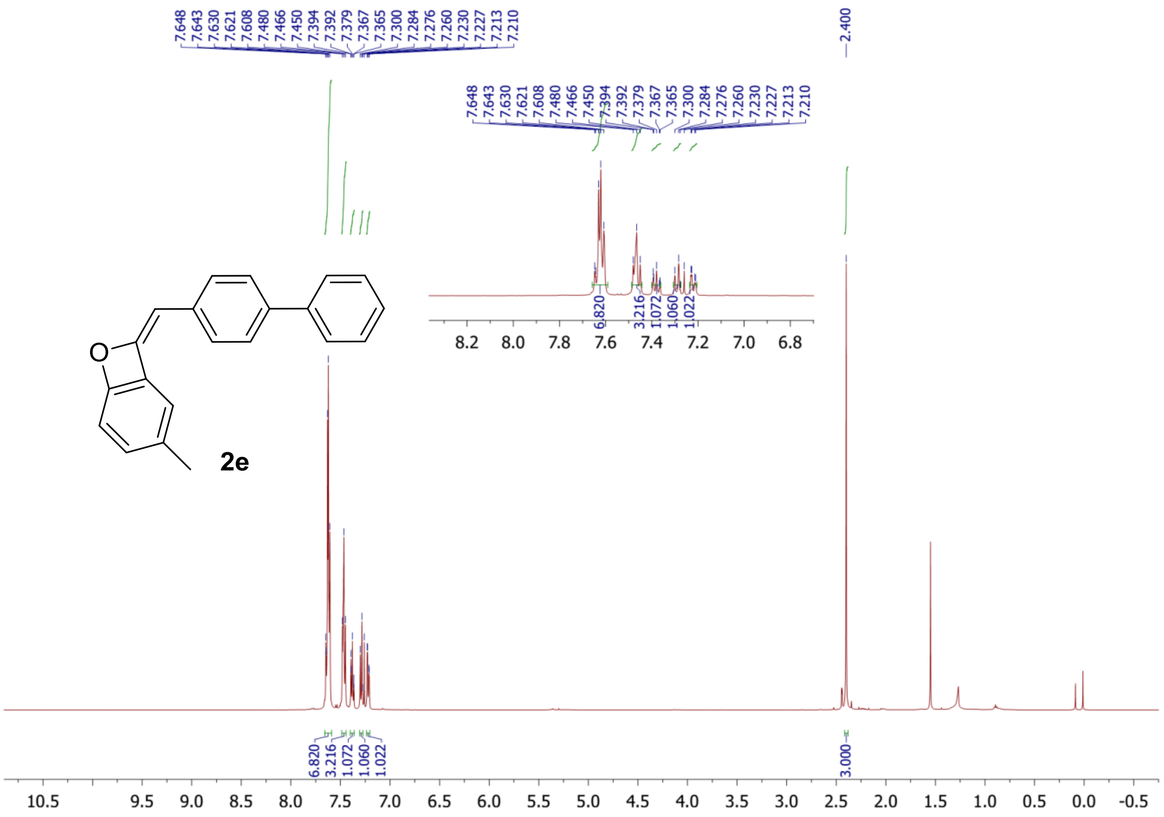

2e, ^{13}C NMR, 126 MHz, $CDCl_3$

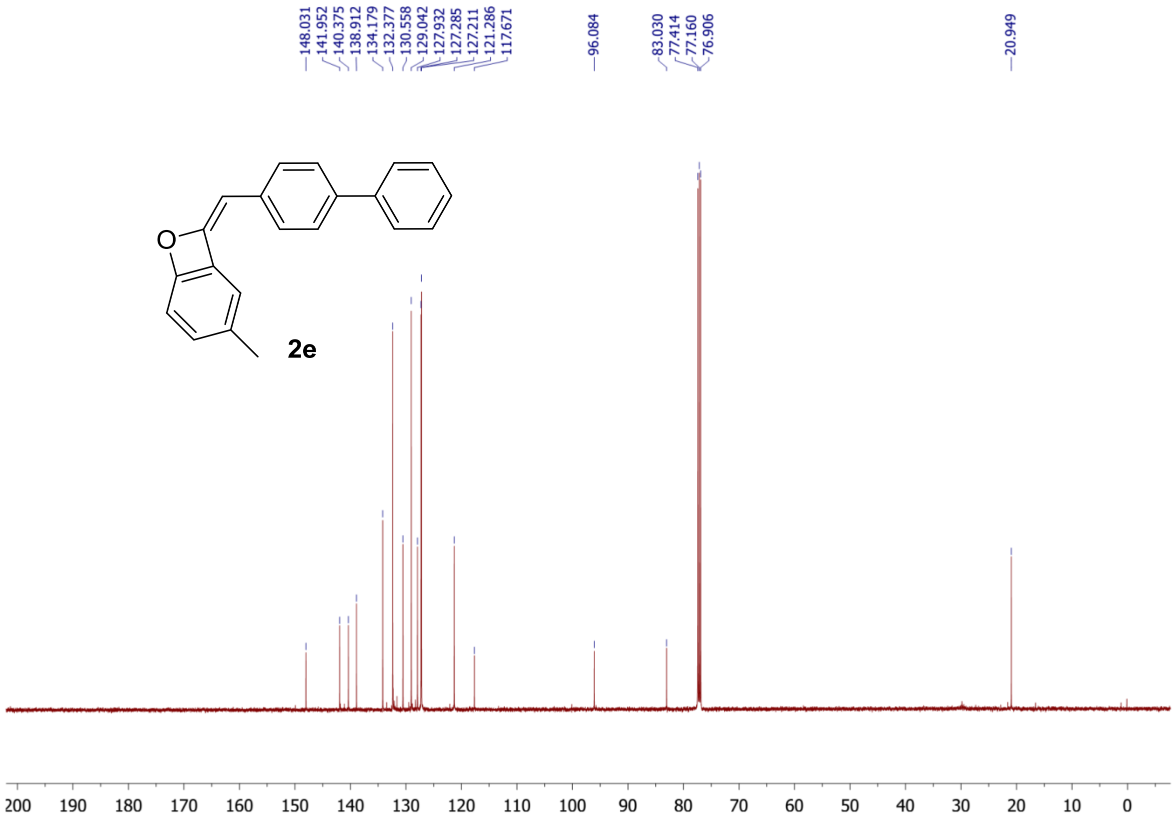

2f, ^{1}H NMR, 500 MHz, $CDCl_3$

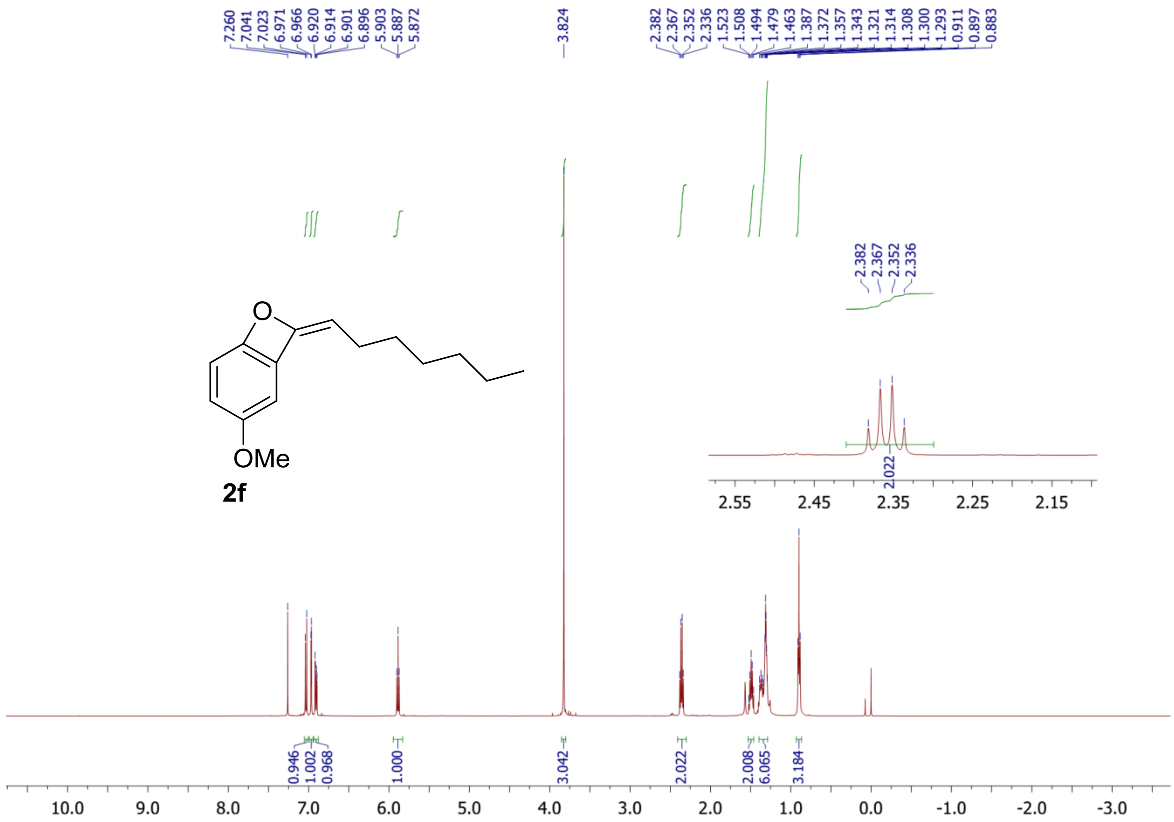

2f, ^{13}C NMR, 126 MHz, $CDCl_3$

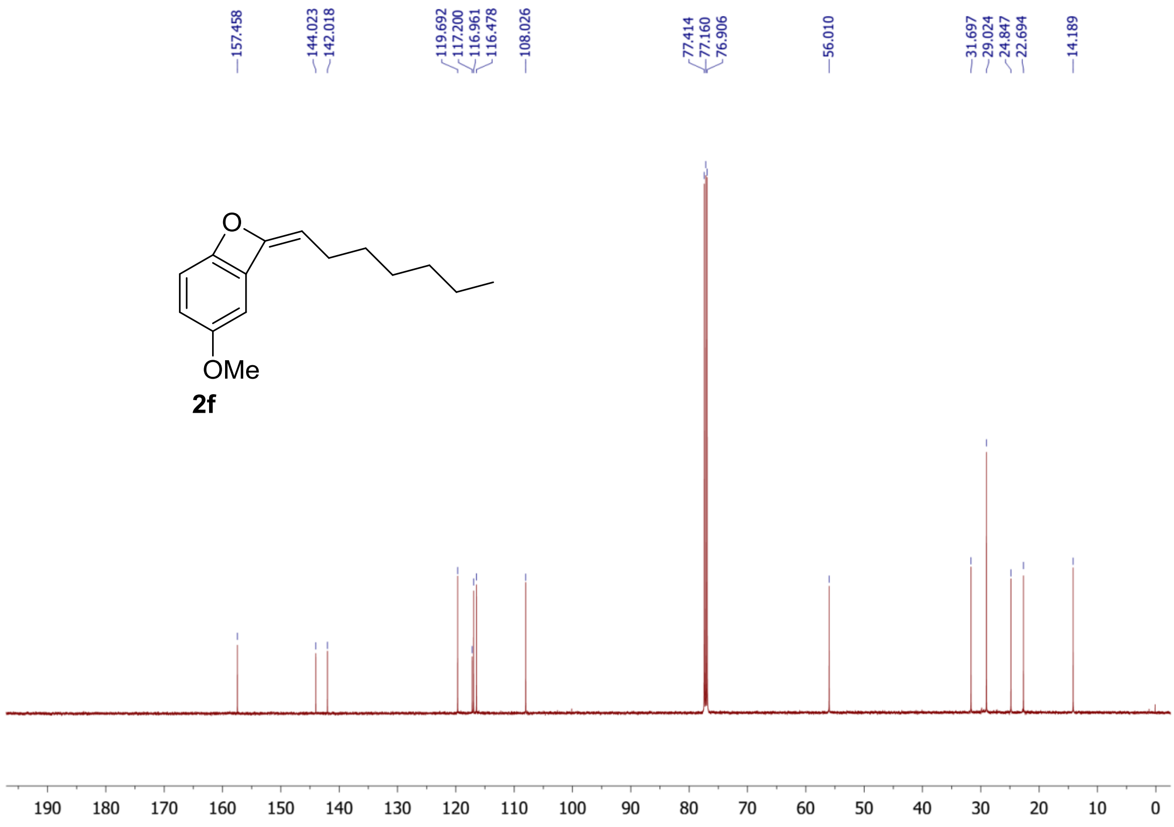

2g, ^{1}H NMR, 500 MHz, $CDCl_3$

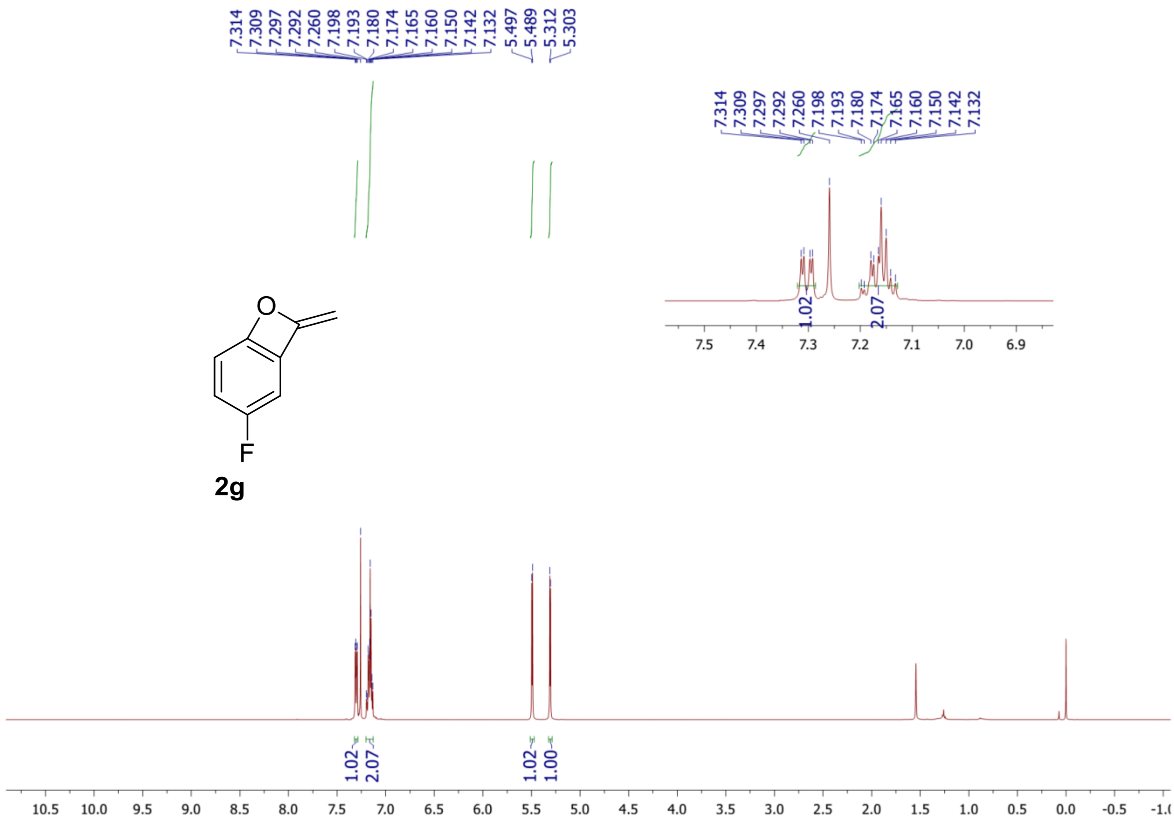

2g, ^{13}C NMR, 126 MHz, $CDCl_3$

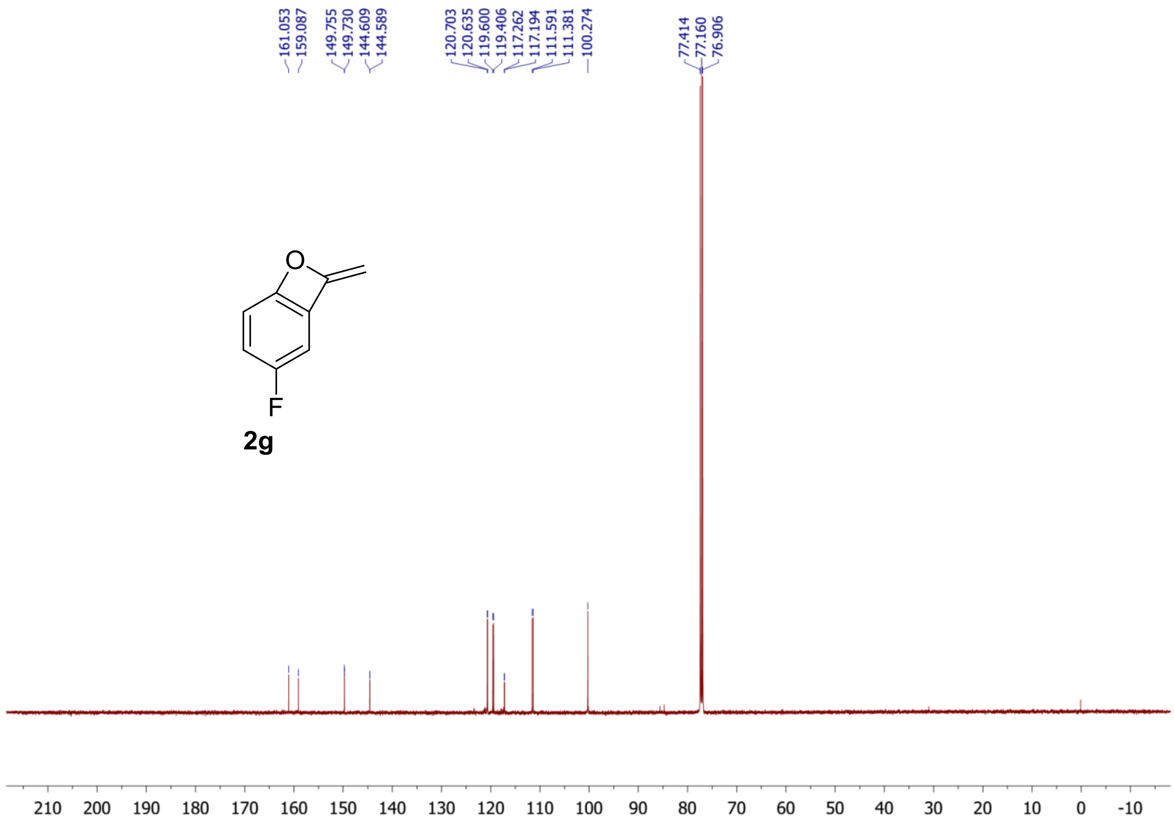

2h, ^{1}H NMR, 500 MHz, $CDCl_3$

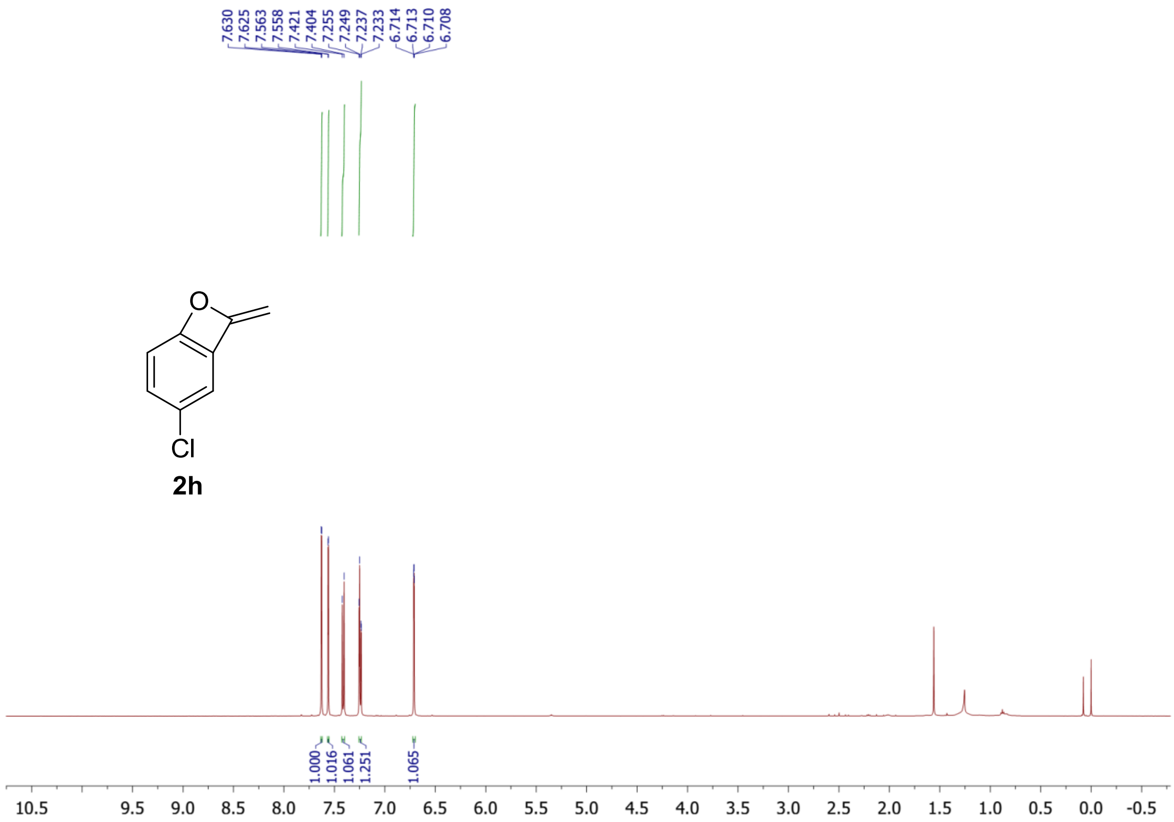

2h, ^{13}C NMR, 126 MHz, $CDCl_3$

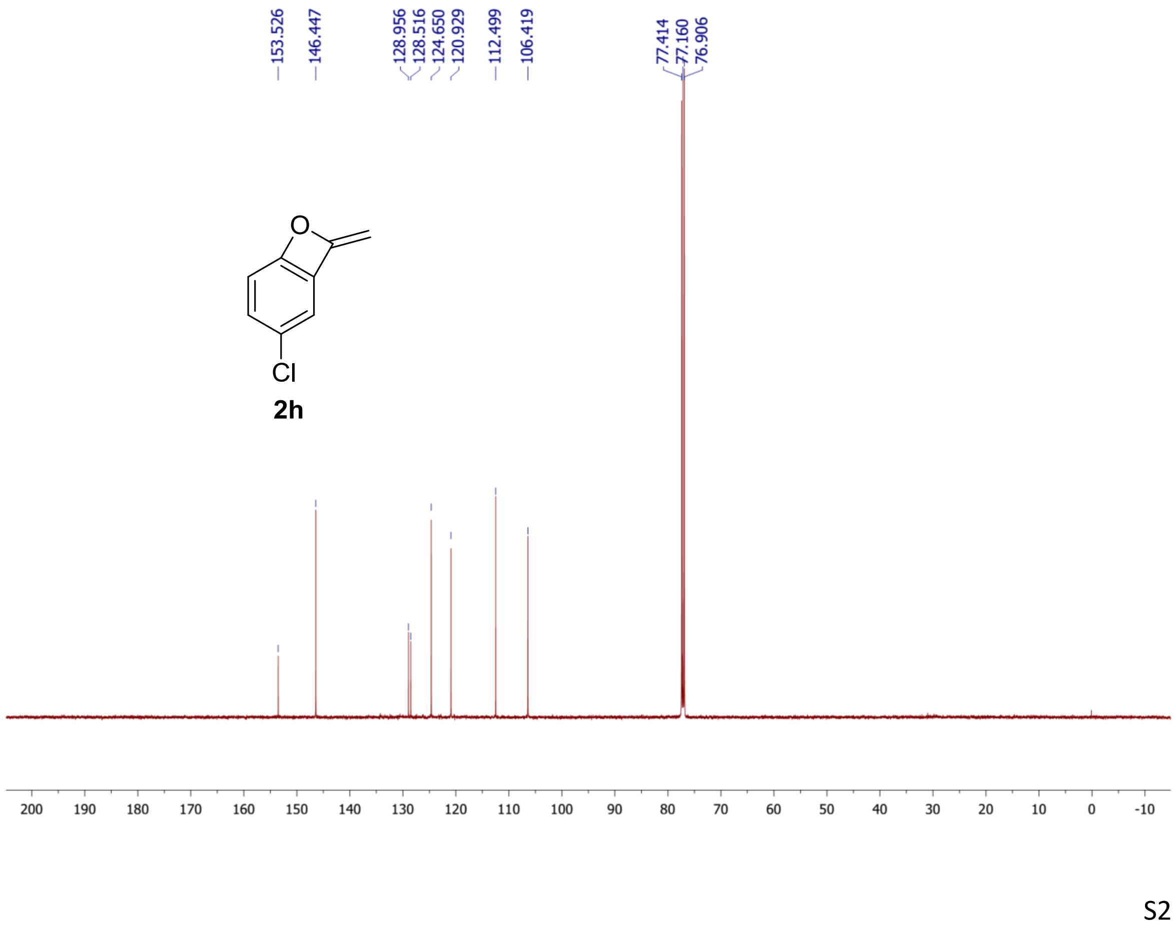

2i, ^{1}H NMR, 500 MHz, $CDCl_3$

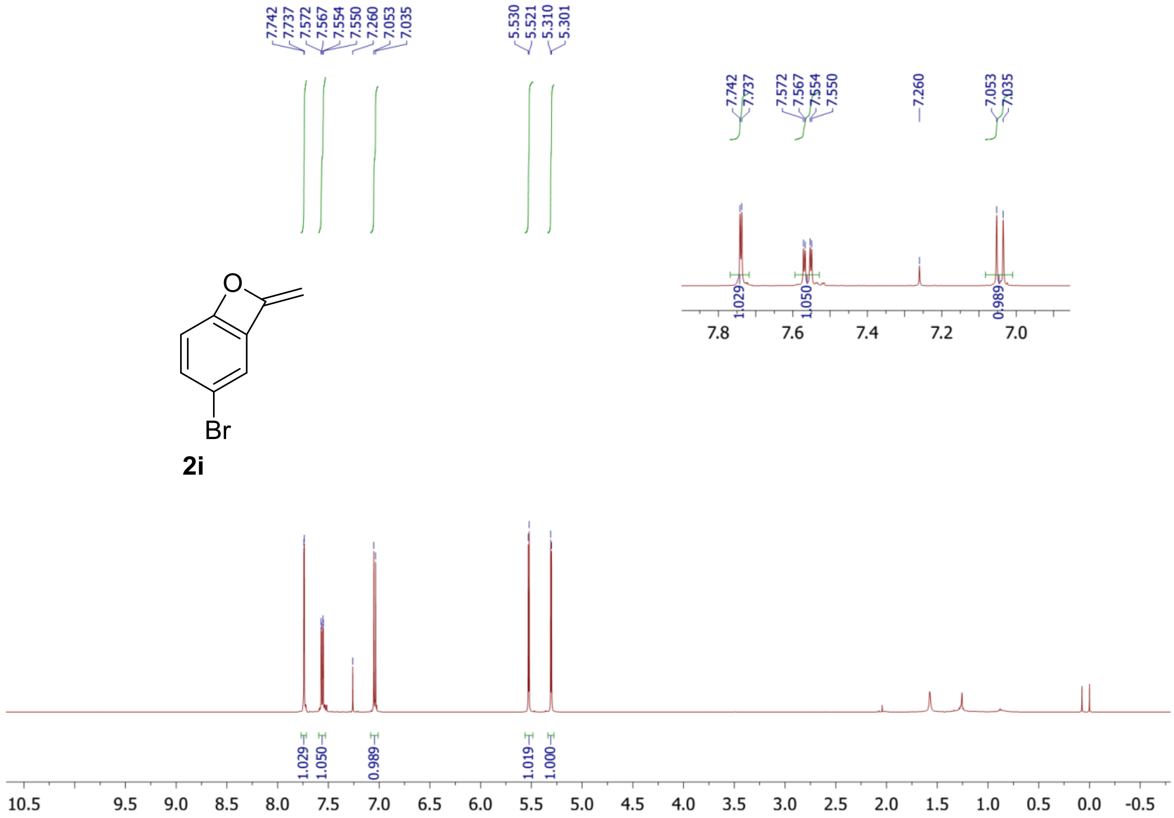

2i, ^{13}C NMR, 126 MHz, $CDCl_3$

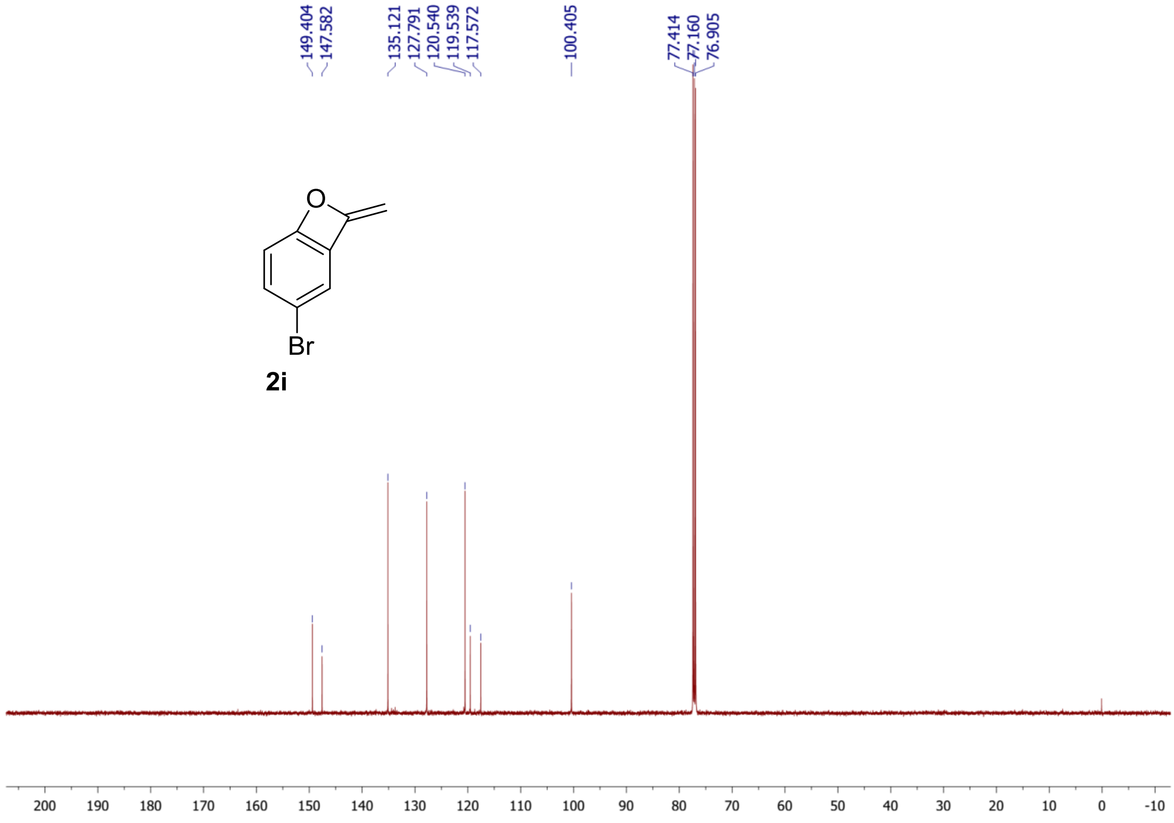

2j, ^{1}H NMR, 500 MHz, $CDCl_3$

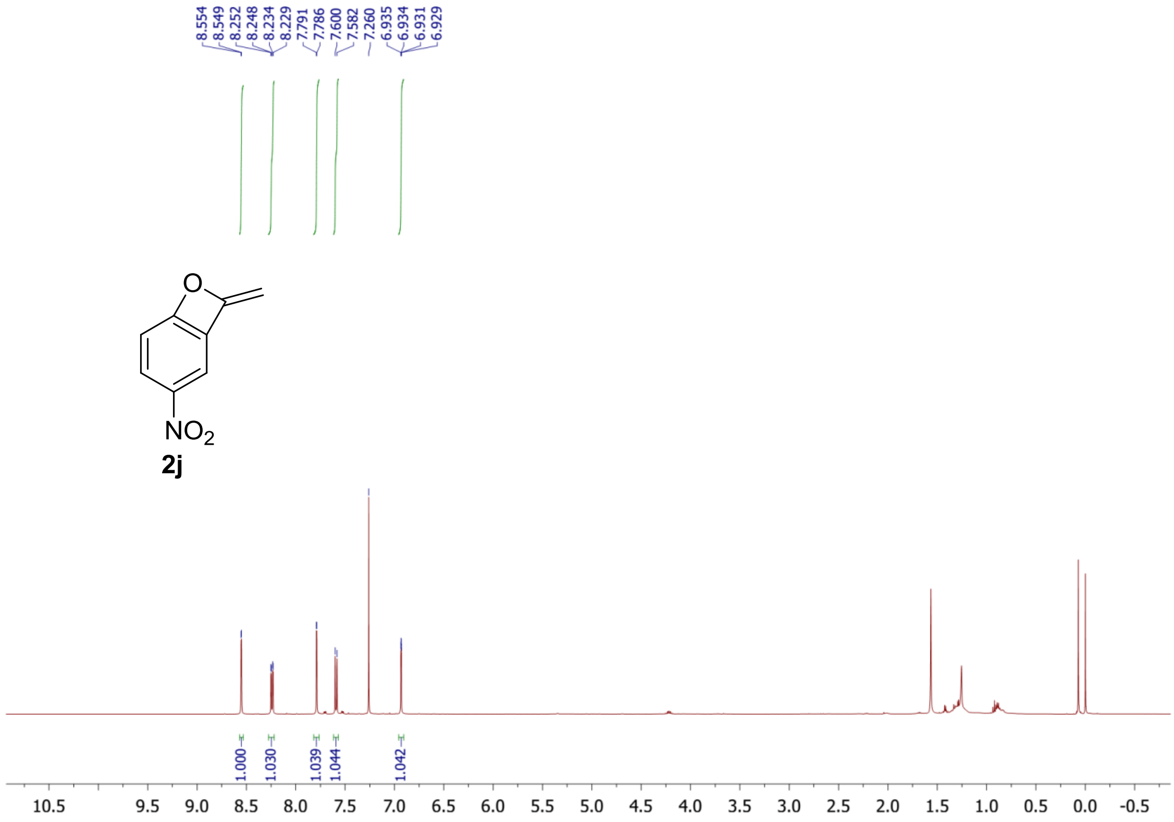

2j, ^{13}C NMR, 126 MHz, $CDCl_3$

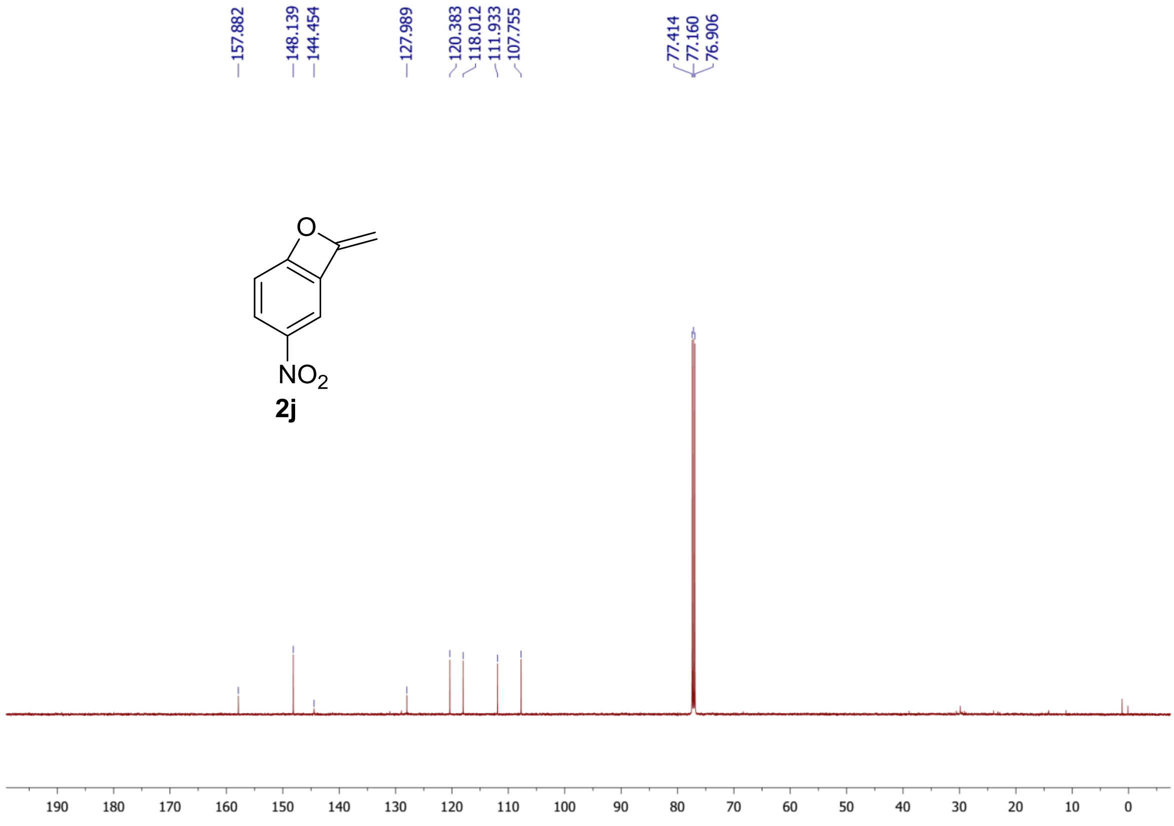

2k, ^{1}H NMR, 500 MHz, $CDCl_3$

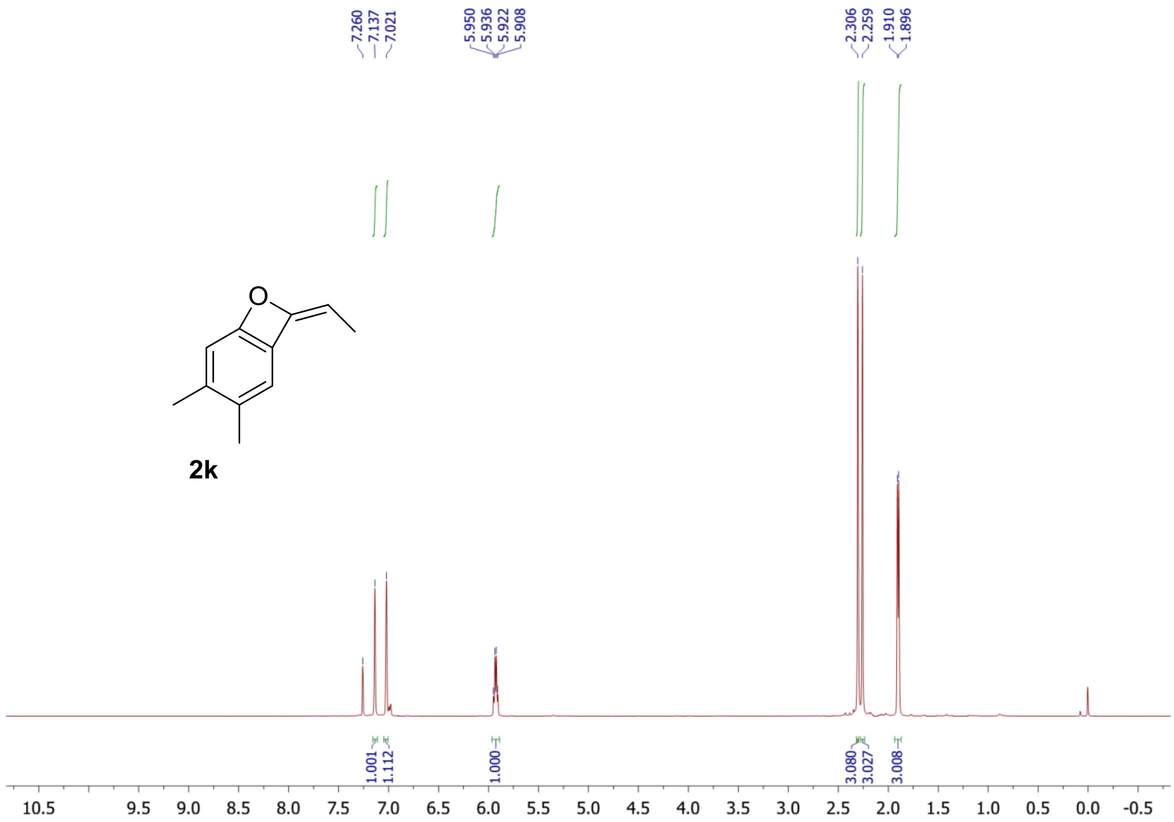

2k, ^{13}C NMR, 126 MHz, $CDCl_3$

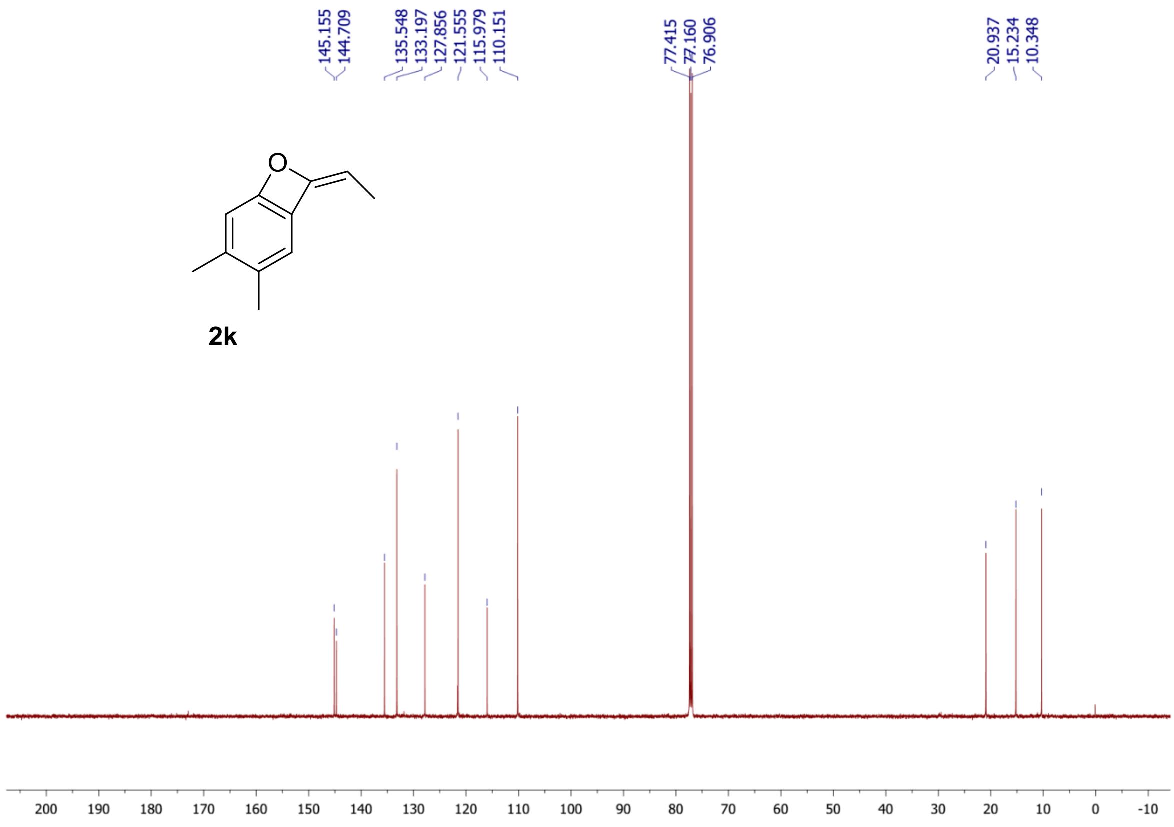

2l, ^{1}H NMR, 500 MHz, $CDCl_3$

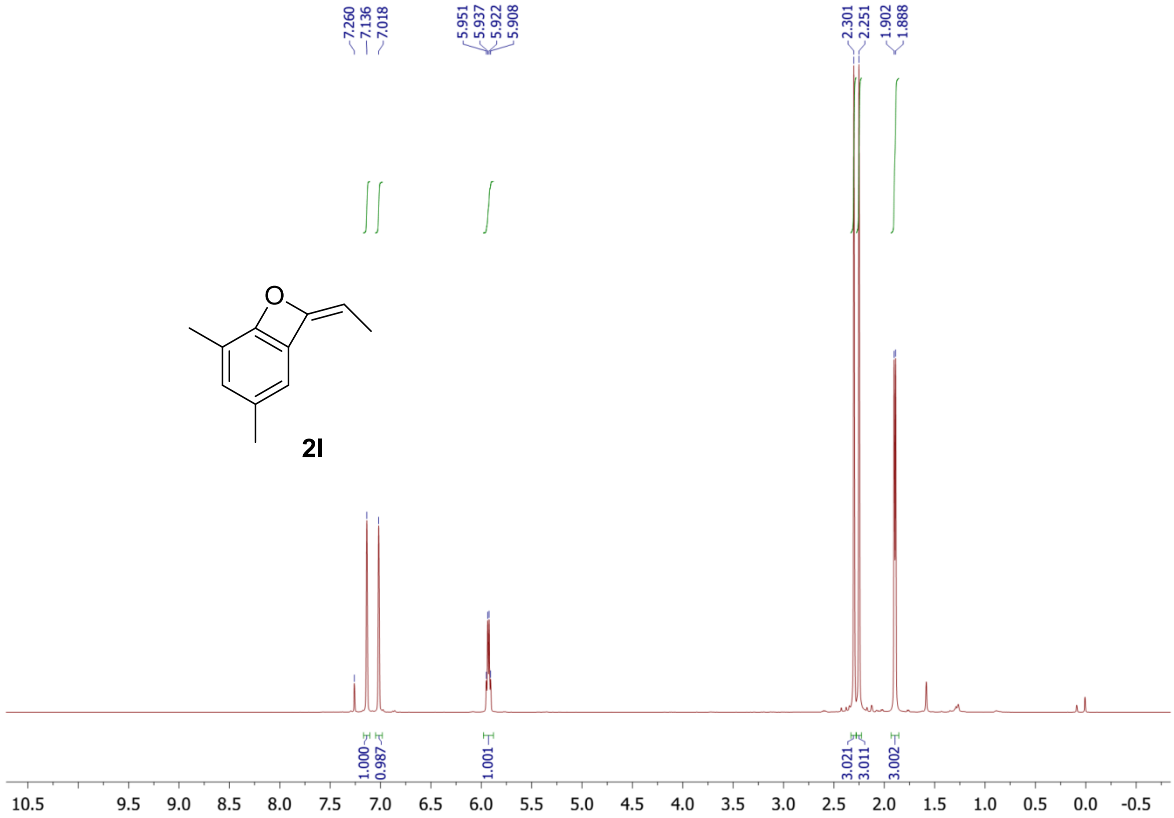

2l, ^{13}C NMR, 126 MHz, $CDCl_3$

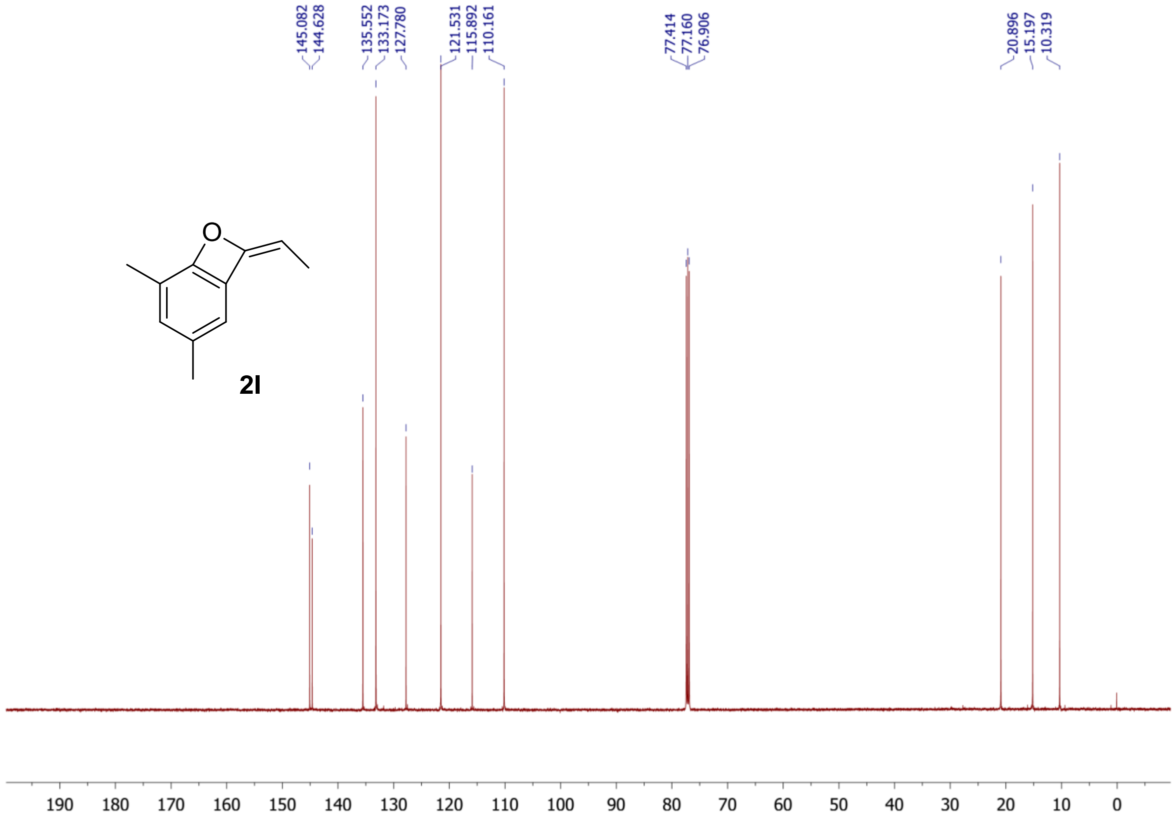

2m, ^{1}H NMR, 500 MHz, $CDCl_3$

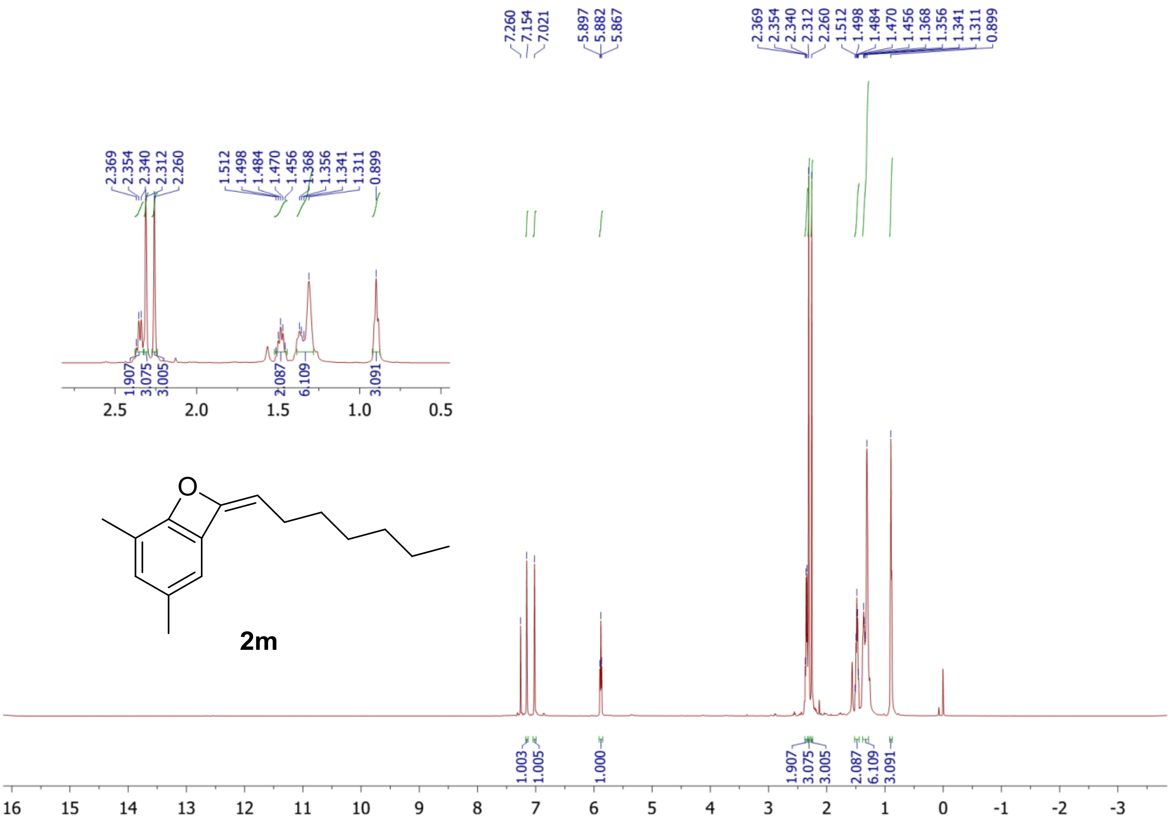

2m, ^{13}C NMR, 126 MHz, $CDCl_3$

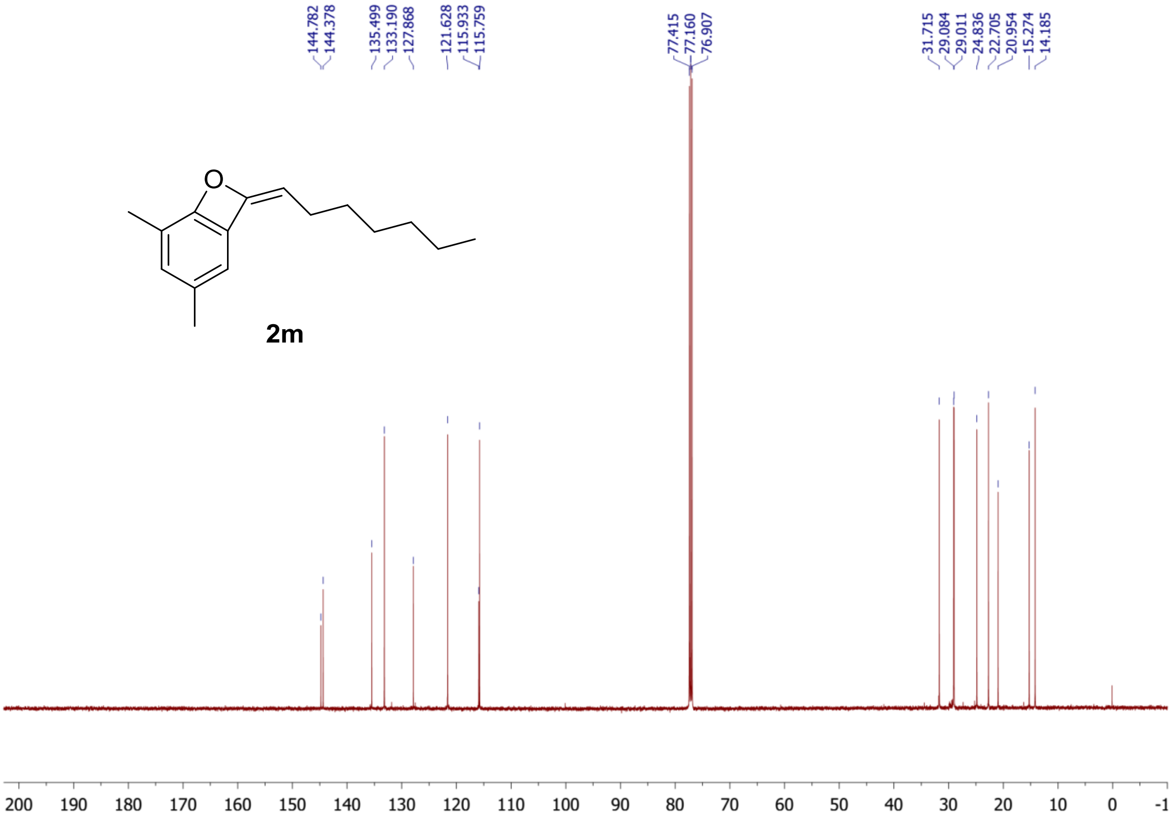

2n, ^{1}H NMR, 500 MHz, $CDCl_3$

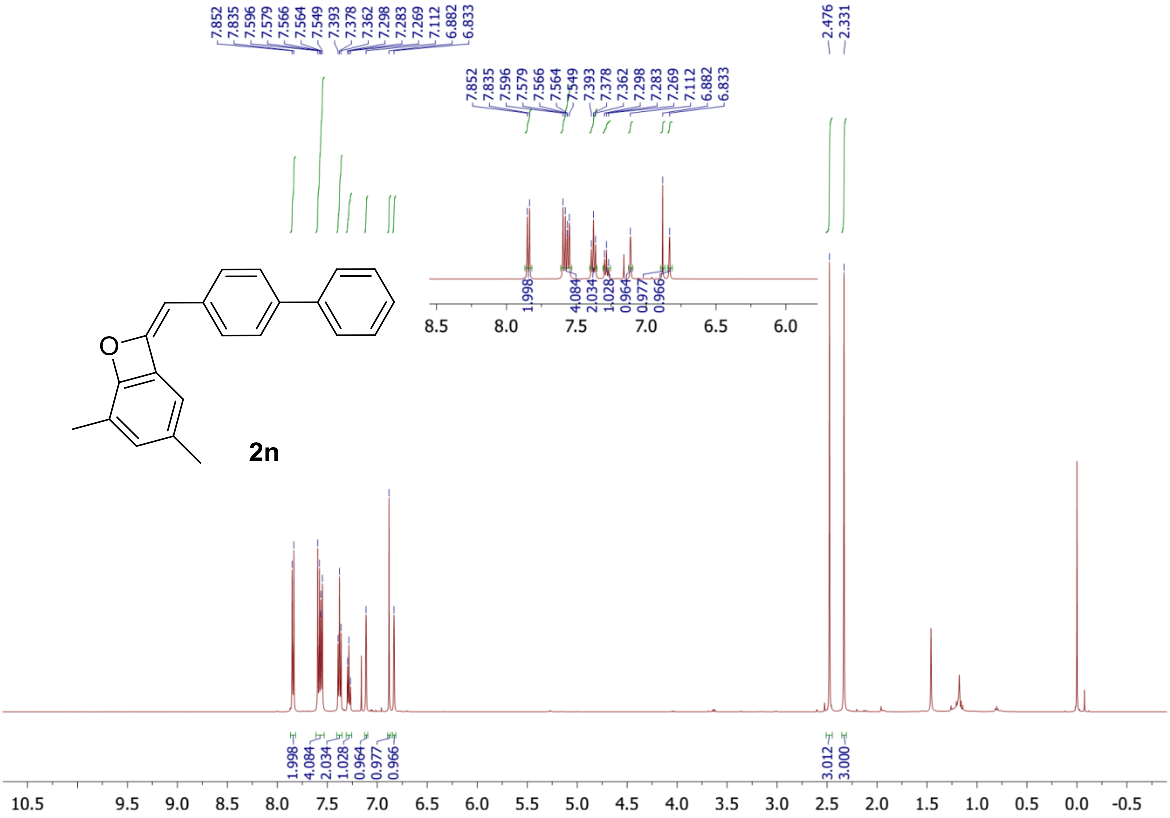

2n, ^{13}C NMR, 126 MHz, $CDCl_3$

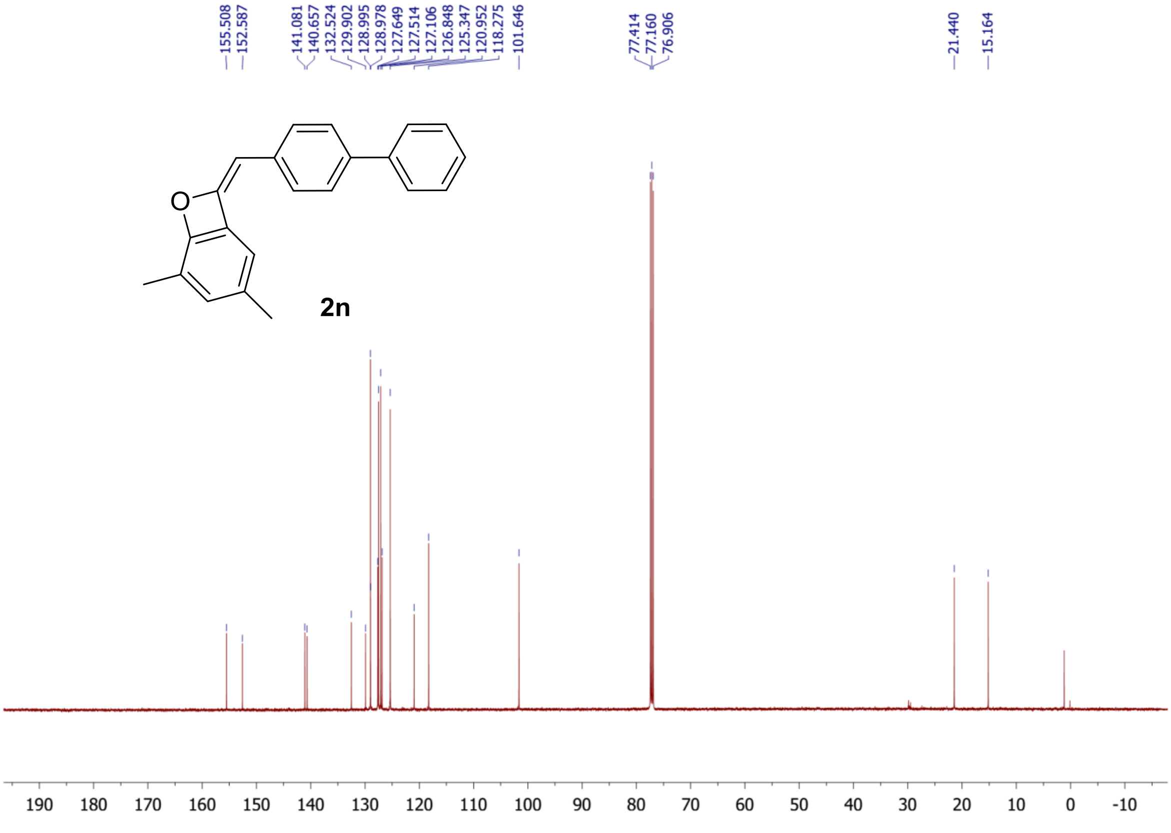

2o, ^{1}H NMR, 500 MHz, $CDCl_3$

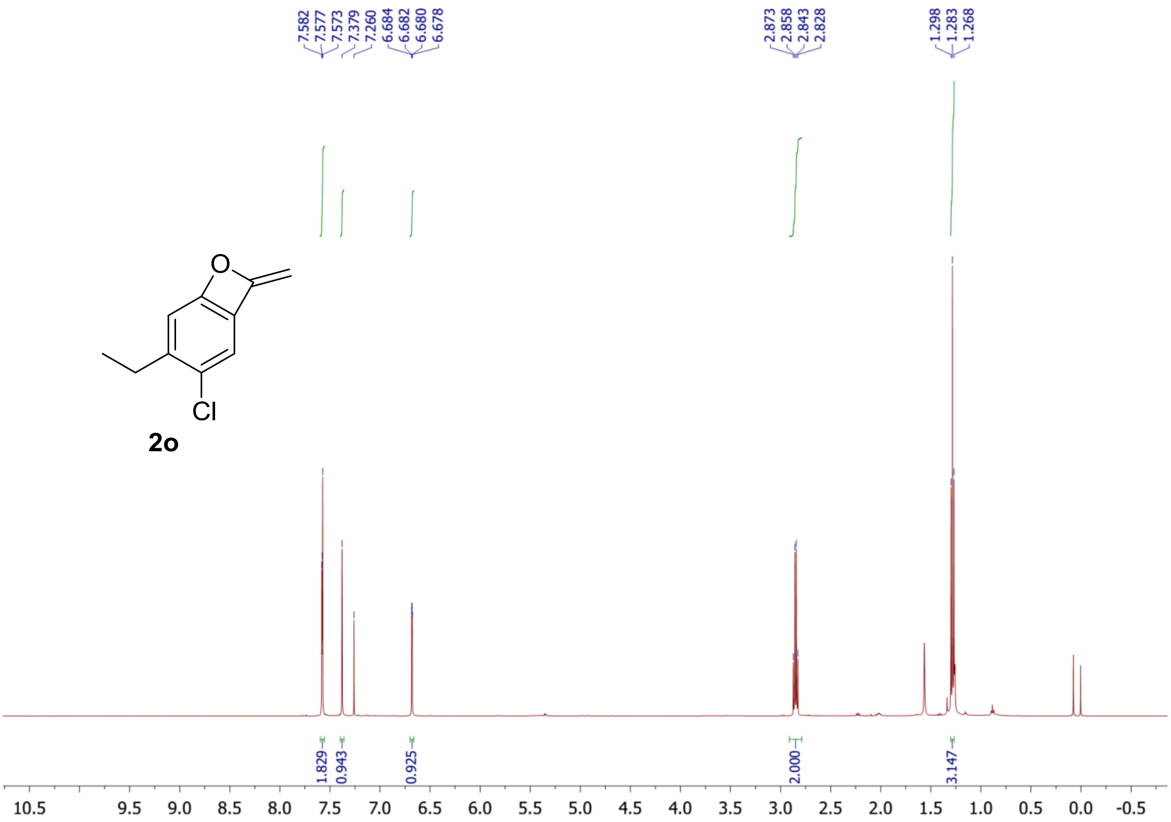

2o, ^{13}C NMR, 126 MHz, $CDCl_3$

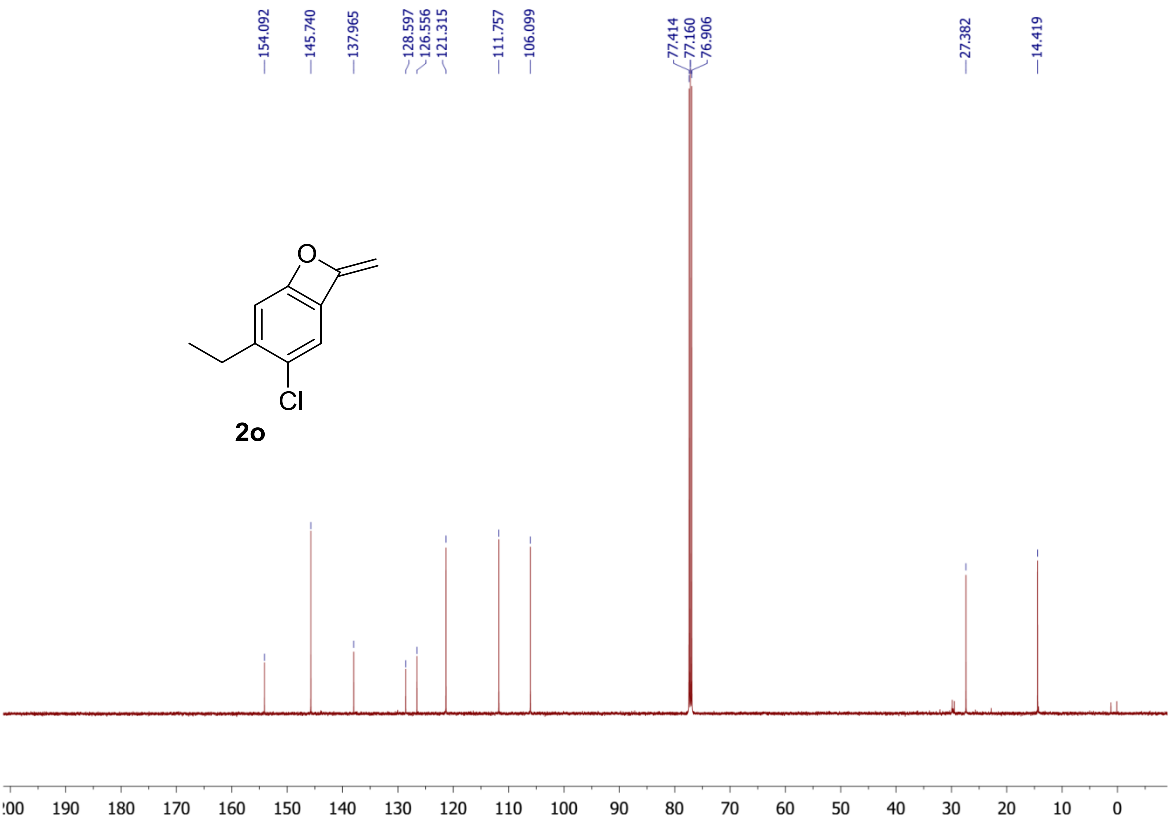

2p, ^{1}H NMR, 500 MHz, $CDCl_3$

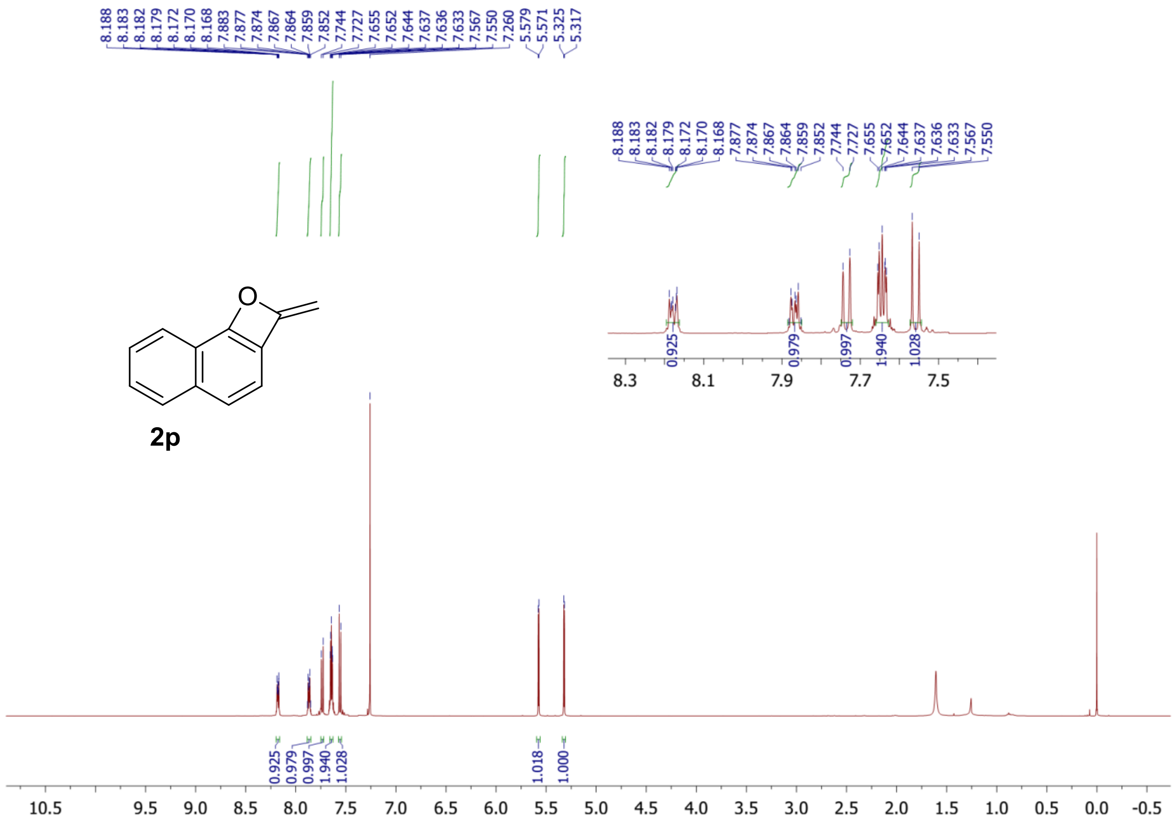

2p, ^{13}C NMR, 126 MHz, $CDCl_3$

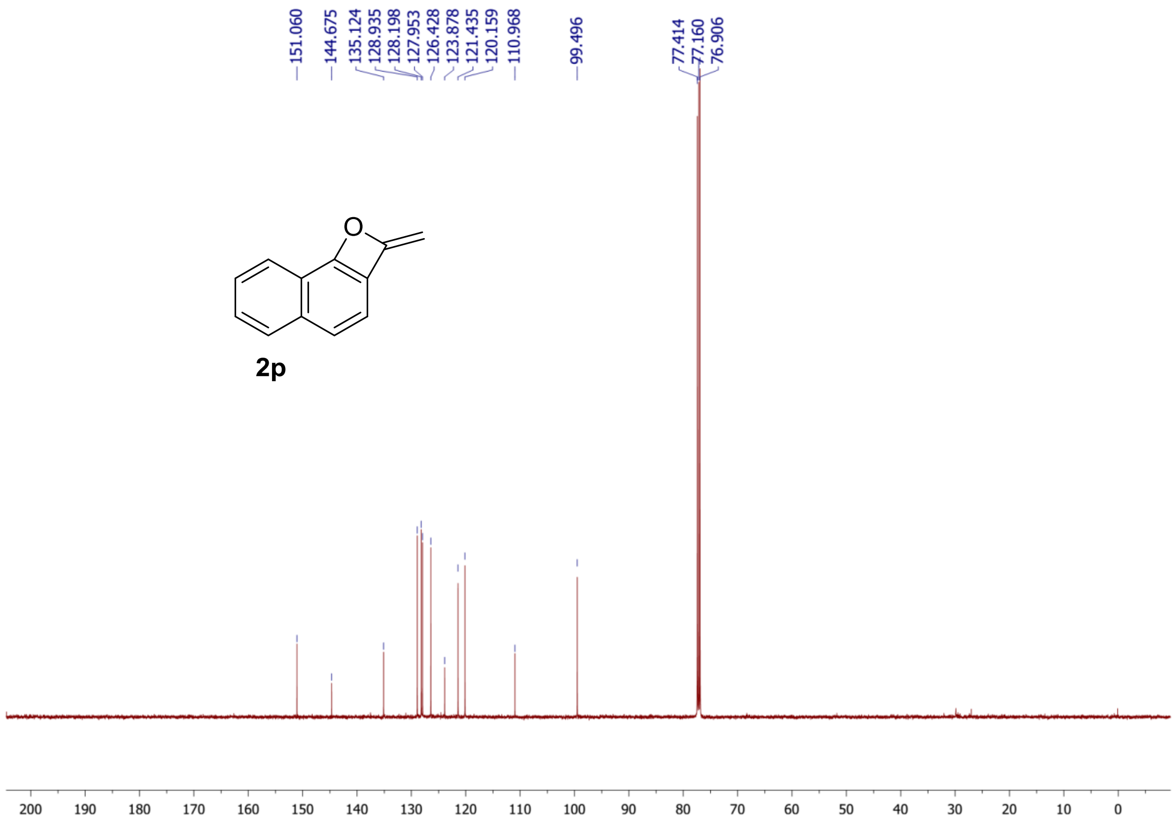

2q, ^{1}H NMR, 500 MHz, $CDCl_3$

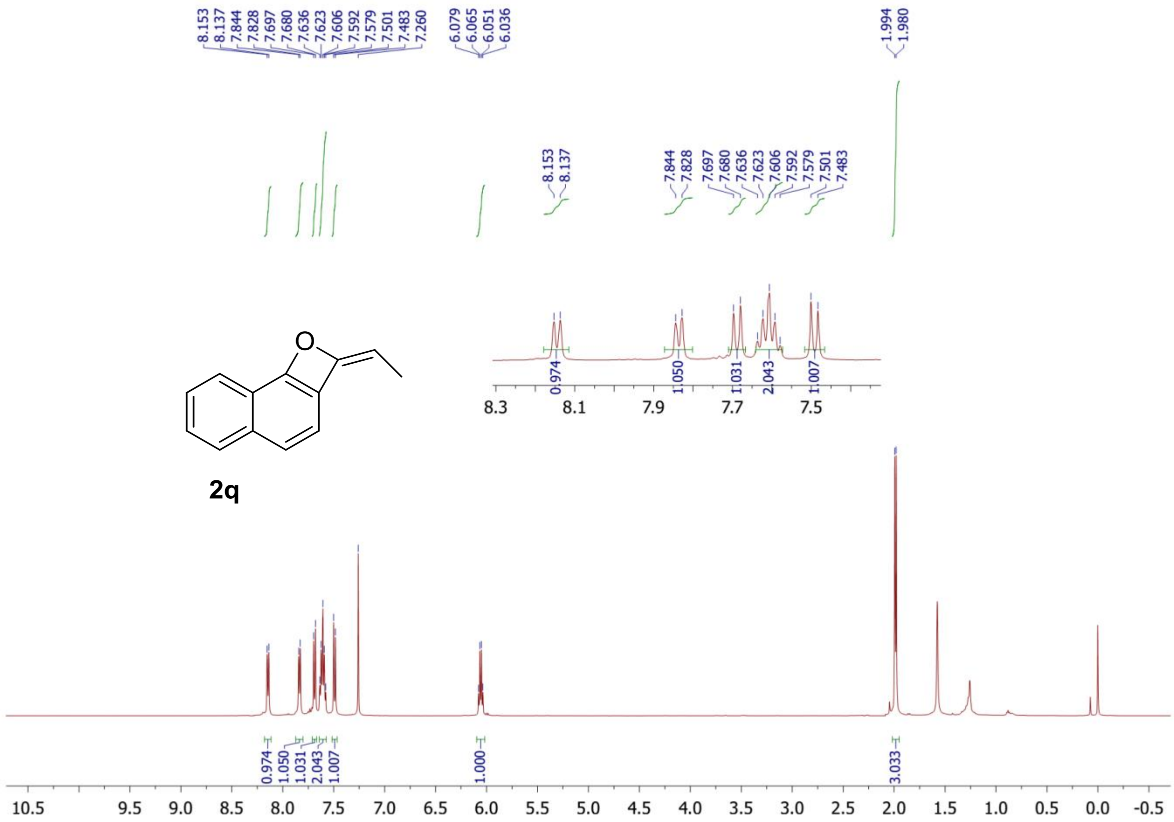

2q, ^{13}C NMR, 126 MHz, $CDCl_3$

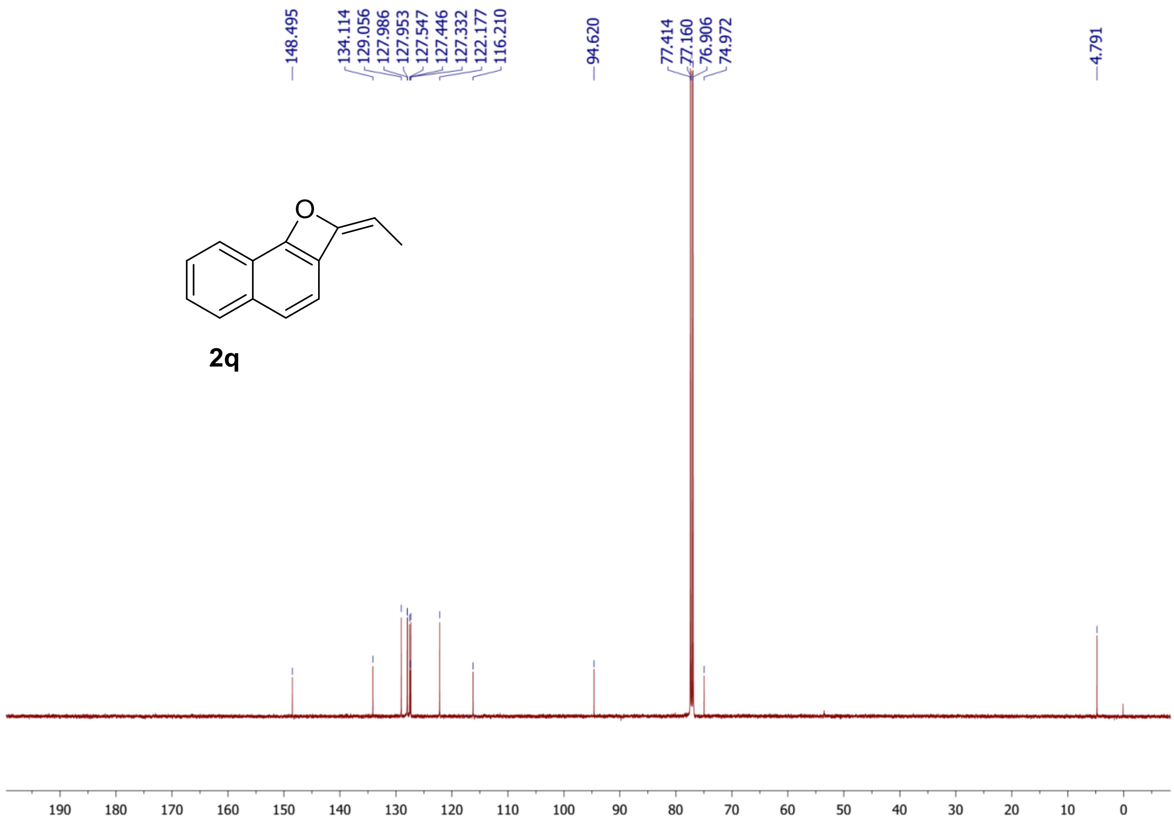

2r, ^{1}H NMR, 500 MHz, $CDCl_3$

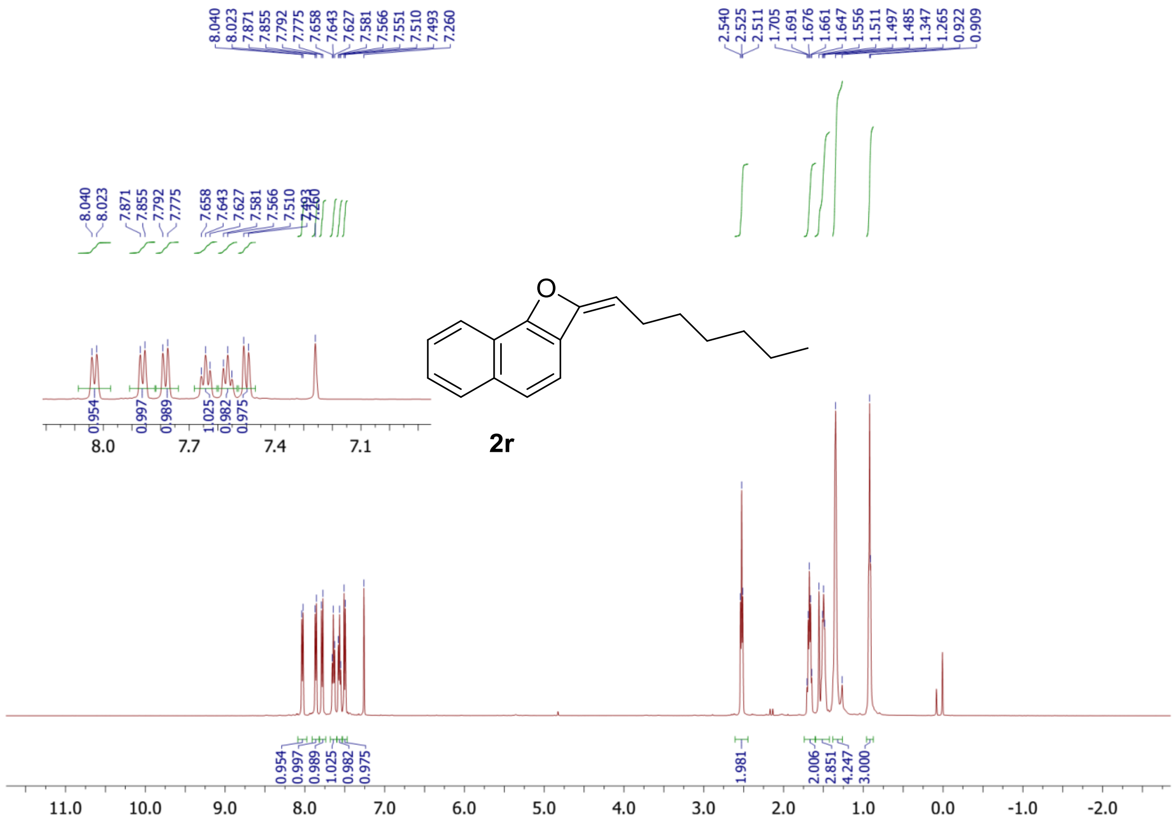

2r, ^{13}C NMR, 126 MHz, $CDCl_3$

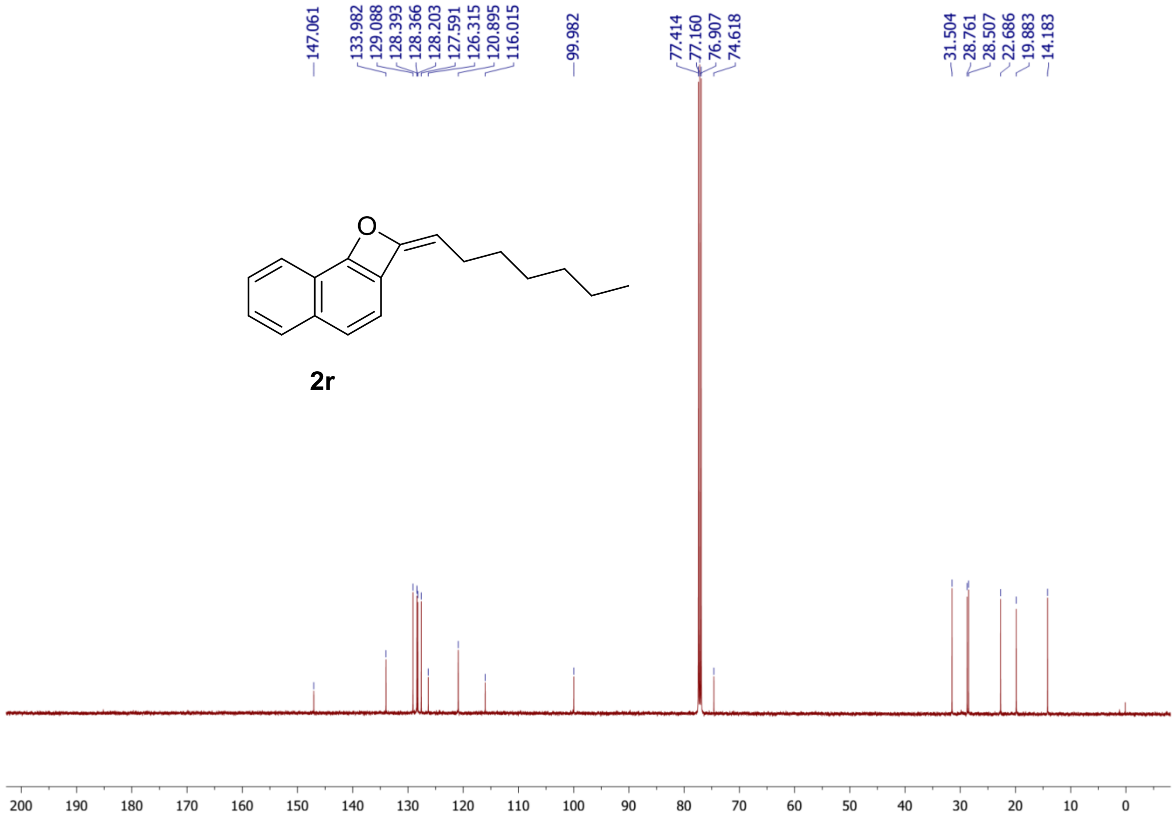

2s, ^{1}H NMR, 500 MHz, $CDCl_3$

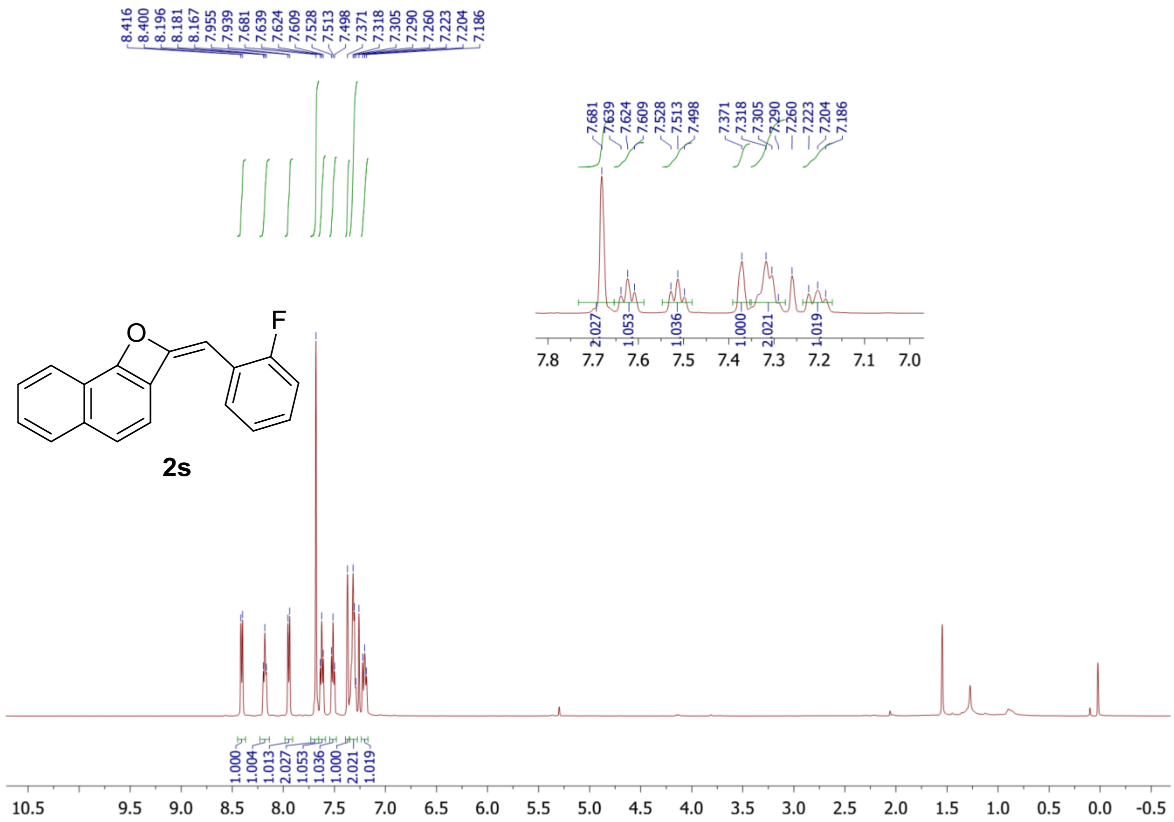

2s, ^{13}C NMR, 126 MHz, $CDCl_3$

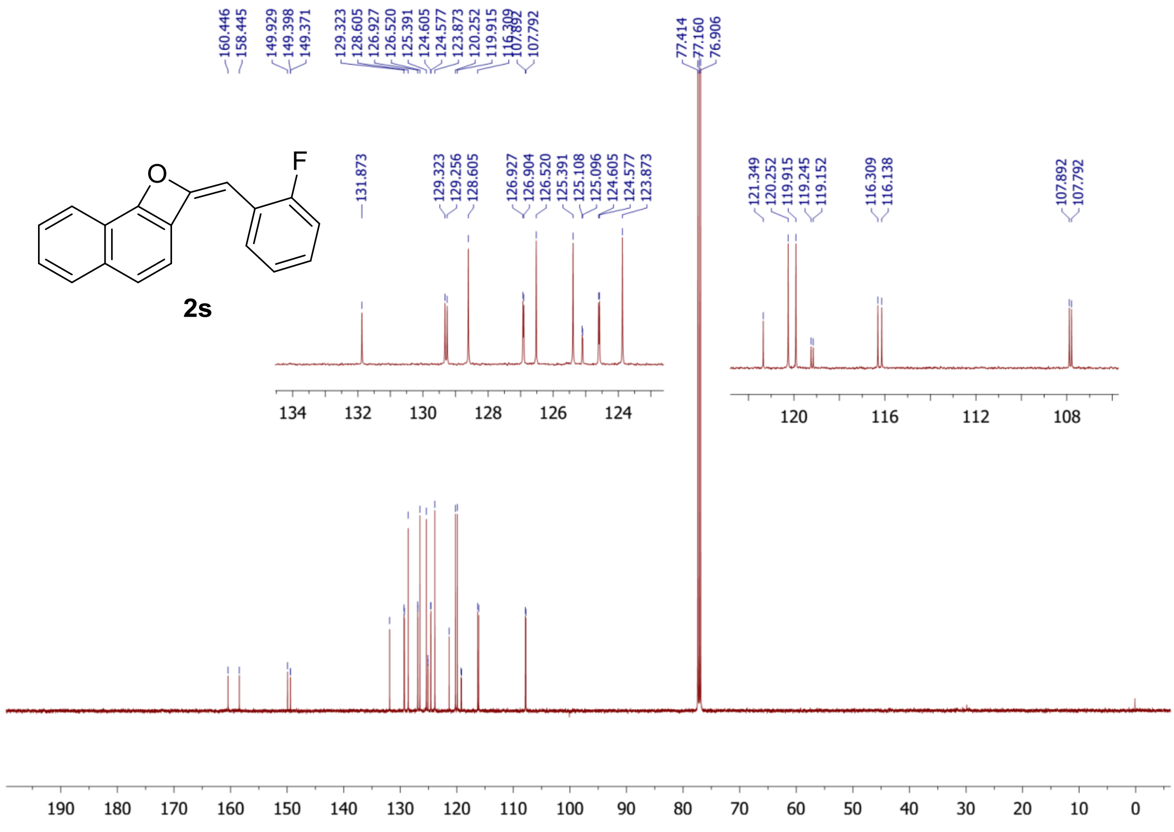

Supplement: File 1 — Conditions optimization, characterization data and copies of NMR spectra. [file Beilstein_J_Org_Chem-15-976-s001.pdf]
